# A microbiota-generated bile salt induces biofilm formation in *Clostridium difficile*

**DOI:** 10.1038/s41522-019-0087-4

**Published:** 2019-05-09

**Authors:** Thomas Dubois, Yannick D. N. Tremblay, Audrey Hamiot, Isabelle Martin-Verstraete, Julien Deschamps, Marc Monot, Romain Briandet, Bruno Dupuy

**Affiliations:** 1 Laboratoire Pathogenèse des Bactéries Anaérobies, Institut Pasteur, Université Paris Diderot, Sorbonne Paris Cité, Paris, France; 2 Institut Micalis, INRA, AgroParisTech, Université Paris-Saclay, Jouy-en-Josas, France; 3 Present Address: INRA, UMR UMET, Villeneuve d’Ascq, France

**Keywords:** Biofilms, Bacteriology, Pathogens

## Abstract

*Clostridium difficile* is a major cause of nosocomial infections. Bacterial persistence in the gut is responsible for infection relapse; sporulation and other unidentified mechanisms contribute to this process. Intestinal bile salts cholate and deoxycholate stimulate spore germination, while deoxycholate kills vegetative cells. Here, we report that sub-lethal concentrations of deoxycholate stimulate biofilm formation, which protects *C*. *difficile* from antimicrobial compounds. The biofilm matrix is composed of extracellular DNA and proteinaceous factors that promote biofilm stability. Transcriptomic analysis indicates that deoxycholate induces metabolic pathways and cell envelope reorganization, and represses toxin and spore production. In support of the transcriptomic analysis, we show that global metabolic regulators and an uncharacterized lipoprotein contribute to deoxycholate-induced biofilm formation. Finally, *Clostridium scindens* enhances biofilm formation of *C. difficile* by converting cholate into deoxycholate. Together, our results suggest that deoxycholate is an intestinal signal that induces *C. difficile* persistence and may increase the risk of relapse.

## Introduction

*Clostridium difficile*, a Gram-positive anaerobic spore-forming bacterium, is the major cause of nosocomial infection associated with antibiotic therapy, with clinical manifestations ranging from mild diarrhea to life-threatening pseudomembranous colitis.^1^ *C. difficile* infection (CDI) is now considered to be a serious threat to the healthcare system and is increasingly recognized as a community-associated infection.^2^ Moreover, CDI is often associated with high recurrence rate (up to 30%) that can be caused either by relapse or reinfection.^3^ *C. difficile* pathogenesis is a multi-step process that begins by the disruption of the healthy microbiota following antibiotic treatments leading to the colonization and toxin production (TcdA and TcdB), which is responsible for the symptoms of CDI.^4^

Ingestion of spores from the environment is the initial step of infection. Once ingested, spores are exposed to various host factors and host-associated stresses. Specifically, conjugated and deconjugated primary bile salts in the small intestine induce germination of spores into metabolically active vegetative cells. Taurocholate (TCA), a conjugated version of cholate (CHO), is the bile salt with the strongest spore germinating activity on *C. difficile*.^5^ Once *C. difficile* reaches the large intestine, vegetative cells are exposed to other host factors and microbiota-generated metabolites, including secondary bile salts such as deoxycholate (DOC) and lithocholate (LCA), which are toxic to vegetative cells. Although spore germination and outgrowth in response to different types of bile acids has been studied,^6^ the response of vegetative cells to bile salts has yet to be fully characterized. Bile salts affect the bacterial cell membrane but can also damage DNA and denature proteins as well as generate several stresses.^7^ However, bile salts can act as signals for pathogenic bacteria to switch lifestyle and induce host colonization or virulence.^8,9^ In *Shigella flexneri*, *Vibrio cholerae*, *Campylobacter jejuni*, and *Listeria monocytogenes*, bile salts also induce biofilm formation.^10–13^

Biofilms are defined as structured communities of microorganisms associated with surfaces and encased in a self-produced extracellular matrix, which varies among bacterial species.^14^ Biofilm formation by *C. difficile* received increased interest in the last decade. *C. difficile* can form biofilms as a single species or with other anaerobic intestinal bacteria on different abiotic surfaces.^15–18^ In addition, *C. difficile* can incorporate a multi-species biofilm formed in a chemostat modeling the human gut^19^ or cell communities in vivo.^20^ Recently, it was shown that *C. difficile* forms a glycan-rich biofilm-like structure in a mono-associated mouse model.^21^ As shown for many pathogens, *C. difficile* cells grown as biofilms are less sensitive to antibiotics commonly used to treat CDI.^17,22^

Several factors, including cell surface components and regulators, have been shown to influence *C. difficile* biofilm formation.^16,18,23–25^ Furthermore, environmental factors such as sub-inhibitory concentrations of metronidazole and vancomycin induce biofilm formation in *C. difficile*.^17,26^ Nevertheless, the biology of biofilm formation by *C. difficile* in the gastrointestinal environment is still poorly characterized.

Based on the importance of bile salts during *C. difficile* colonization, we investigated the role of various bile salts on the induction of biofilm formation and we demonstrated that the secondary bile salt DOC has the most significant effect. Gene expression analysis of biofilm cells grown in the presence of DOC suggests that bile salt signaling may play an important role in the mechanism involved in survival and persistence of *C. difficile* in the dysbiotic intestinal environment. These results contribute to the understanding of the relationship between *C. difficile* and the host environment during infection.

## Results

### Specific bile salts induce *C. difficile* biofilm formation

**Fig. 1:**
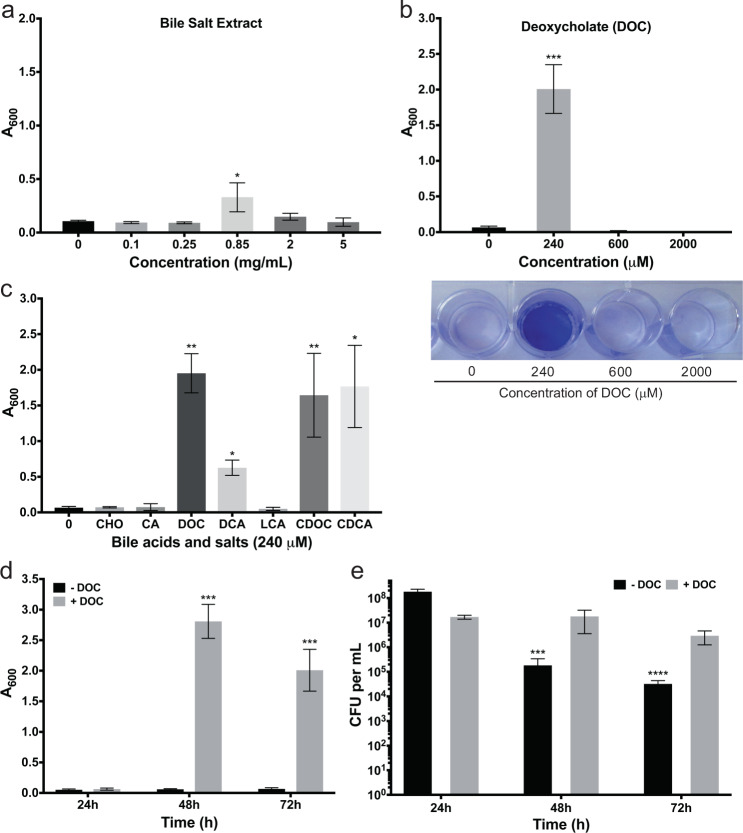
Effects of bile salts and deoxycholate on biofilm formation by *C. difficile* strain 630Δ*erm*. **a** Bacteria were grown in BHISG supplemented with the indicated concentrations of bile salt extract or **b** deoxycholate. **a**, **b** Biofilm formation was evaluated at 72 h. In (**b**), representative image of a CV stained biofilm produced by cells cultured in the presence of increasing DOC concentrations. **c** Biofilm formation was evaluated after 48 h growth in the presence of 240 µM bile acids [cholic acid (CA), deoxycholic acid (DCA), lithocholic acid (LCA), chenodexycholic acid (CDCA)] or bile salts [cholate (CHO), deoxycholate (DOC) or chenodeoxycholate (CDOC)]. Asterisks indicate statistical significance determined with a Kruskal–Wallis test followed by an uncorrected Dunn’s test (*p ≤ 0.05, **p ≤ 0.001, ***p ≤ 0.001 vs BHISG with 0 mg/mL bile salts or 0 µM DOC). **d** Biofilm formation kinetics in the presence or absence of 240 µM DOC. Biofilm formation (**a**–**d**) was measured using a crystal violet assay that included two PBS washing before staining (see the Methods section). **e** Kinetics of CFU grown in the presence or absence of 240 µM DOC. The CFU counts were performed with unwashed biofilms (see the Methods section). Asterisks indicate statistical significance determined with a two-way ANOVA followed by a Fisher LSD test (***p ≤ 0.001, ****p ≤ 0.0001 vs 24 h). Data shown indicate the mean and the error bars represent the standard error of the mean of at least five experiments performed on different days

To determine the impact of bile salts on biofilm formation by *C. difficile*, we added a commercial bile salt extract (Supplementary Table 1) to supplemented brain–heart infusion with glucose (BHISG). The bile salt extract significantly induced biofilm formation that was resistant to PBS washes at a concentration of 0.85 mg/mL (Fig. 1a). This bile salt extract contains different forms of bile acids and salts. Typically, bile acids are the synthetic form and become bile salts under physiological pH. All primary and secondary bile acids are conjugated prior to secretion by either glycine or taurine and these conjugated bile acids are often referred to as bile salts. Unlike the unconjugated bile acids, which are in the water insoluble HA form (deprotonated), the conjugated bile acids (or bile salts) are mainly in the water-soluble deprotonated form (A-). To identify the specific bile salts responsible for this induction, we then tested a range of concentration of individual bile salts found in the human intestine (Supplementary Table 1).

The concentrations used were selected based on those typically found in human intestine.^6,27^ The primary bile salt chenodeoxycholate (CDOC), and its glycine and taurine conjugates (glycochenodeoxycholate and taurochenodeoxycholate), significantly induced biofilm formation while the secondary bile salt LCA, a CDOC derivative, had no effect (Supplementary Fig. 1a and 1b). The primary bile salt cholate (CHO) or its glycine and taurine conjugates (glycocholate and taurocholate) had no effect on biofilm formation (Supplementary Fig. 1b and 1c). By contrast, the secondary bile salt deoxycholate (DOC), a CHO derivative, strongly induced biofilm formation at 240 µM (Fig. 1b), while the glycine and taurine conjugates of DOC (glycodeoxycholate and taurodeoxycholate) did not (Supplementary Fig. 1d). For both CDOC and DOC, the corresponding acid forms (CDCA and DCA, respectively) have the same impact and the inducing concentration had a weak effect on growth (Fig. 1c and Supplementary Fig. 2). Furthermore, controls with the diluents without bile acids did not have significant effect on growth or biofilm formation. Since DOC is more prevalent than CDOC at the site of infection of *C. difficile*, we decided to characterize biofilm formation induced by DOC. The minimum and maximum concentration of DOC for biofilm induction was 240 and 480 µM, respectively, while the biofilm is induced later (>72 h) when using 480 µM. At the 240 µM concentration, the DOC-induced biofilm reached its maximum at 48 h and decreased slightly at 72 h (Fig. 1d). In addition, the colony-forming units (CFUs) of the unwashed biofilms remain stable in the presence of DOC but decrease over time in the absence of DOC (Fig. 1e). This suggests that cell viability is higher in the DOC-induced biofilms and 48 h was selected as the time for the subsequent analyses.

To ensure that biofilm-induction by DOC was not strain-specific, the effect of DOC was tested on several clinical isolates (Supplementary Table 2) and we showed that DOC significantly induced the formation of biofilm in every strain with the exception of the strain E25, which is a strong-biofilm former in the absence of DOC (Supplementary Fig. 3). Taken together, the data show that DOC strongly and specifically induces the formation of biofilm in *C. difficile*.

### Fermentable sugars potentiate biofilm induction by DOC

Since glucose was added to the medium used for our biofilm assay, we tested whether induction of biofilm by DOC was dependent on the presence of glucose. When glucose was omitted from BHIS, we found that the presence of glucose was essential for DOC to induce biofilm formation (*p* ≤ 0.0001; Supplementary Fig. 4a). When we added different sugars to BHIS, we observed that ribose, fructose and, to a lesser extent, xylose also significantly enhanced the effect of DOC (*p* ≤ 0.0001) while cellobiose, maltose, arabinose, galactose, and sorbitol had no significant effect (Supplementary Fig. 4a). Interestingly, sugars that potentiate the effect of DOC on biofilm formation are fermented by *C. difficile* strains, while those having no effect are not fermented, with the exception of sorbitol.^28^ Since biofilm was only induced when DOC and a fermentable carbon source were added, we concluded that both are required to trigger biofilm formation in *C. difficile*. Given that supplementation of the growth medium with fermentable carbon will result in a drop of pH to 6 during growth,^29^ we buffered BHISG with 50 mM HEPES or MOPS and this abolished biofilm formation (Supplementary Fig. 4b). In the absence of glucose supplementation, the pH was stable at 6.9 despite the presence of 11 mM glucose in the commercial BHI used in our study. Taken together, these results suggested that the catabolism of fermentable sugar lowers the pH of the medium during growth and this might induce biofilm formation with DOC.

### Effect of DOC on the biofilm architecture and population

**Fig. 2:**
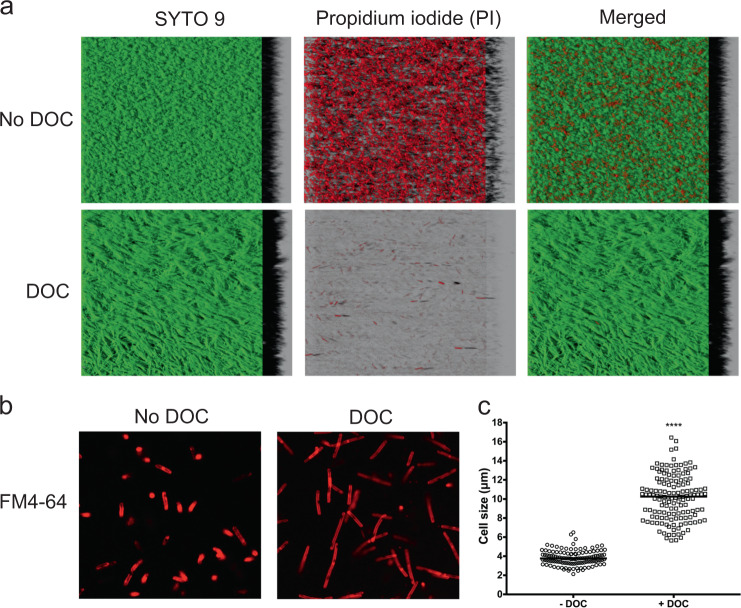
Characterization of the biofilm architecture and population in the presence of DOC. **a** CLSM images of *C. difficile* biofilms formed in the absence or presence of 240 µM DOC stained with Syto 9 (green/living) and propidium iodide (red/dead). **b** CLSM images of *C. difficile* grown in the absence or presence of 240 µM DOC and stained with FM4-64. **a**, **b** The CLSM analyses were performed with unwashed biofilms. **c** Cell length (µm) distribution within a population grown in BHISG or BHIG with 240 µM DOC. Asterisks indicate statistical significance determined with a two-tail Mann–Whitney test (****p ≤ 0.0001 vs BHISG without DOC)

In the presence of DOC, bacteria formed a film with vein-like structure that was tightly attached to the substrate and was resistant to washing with PBS (Supplementary Fig. 5). Additionally, cells formed strong and sticky aggregates that were difficult to resuspend when biofilms had to be detached for downstream analysis. This differs from the structure produced in BHISG in the absence of DOC, which is poorly cohesive and easily detached from the abiotic substrate. We examined by confocal laser scanning microscopy (CLSM) the architecture of the biofilms in the presence or absence of DOC by staining with SYTO9 and propidium iodide dyes used to detect live/dead cells. Given that the biofilms produced in the absence of DOC is easily detachable, all CLSM experiments were done with unwashed biofilm to be able to compare DOC-induced biofilms with untreated biofilms. Consistent with CFU counts, we observed that biofilms formed in the absence of DOC had a larger dead population (red) than those induced by DOC which appears to be mostly composed of live cells (green cells; Fig. 2a). Furthermore, the DOC-induced biofilm forms a continuous lawn of cells that is denser than the non-induced biofilm, which is a structure with craters (Fig. 2a). Additionally, we noticed that *C. difficile* cells in the DOC-induced biofilm increased in length. To confirm this observation, cells from both biofilms were suspended in PBS and stained with the red fluorescent dye FM4-64, which labels cell membranes. CLSM analysis showed an increase in length for cells grown with DOC (Fig. 2b). Specifically, cell length increased from a median of 3.8 μm (±0.8) for those grown in BHISG to a median of 10.3 μm (±2.4) for cells grown with DOC (Fig. 2c). The presence of DOC significantly increased the median size of the cells by 2.7-fold.

### Assembly and stability of the DOC-induced biofilm

**Fig. 3:**
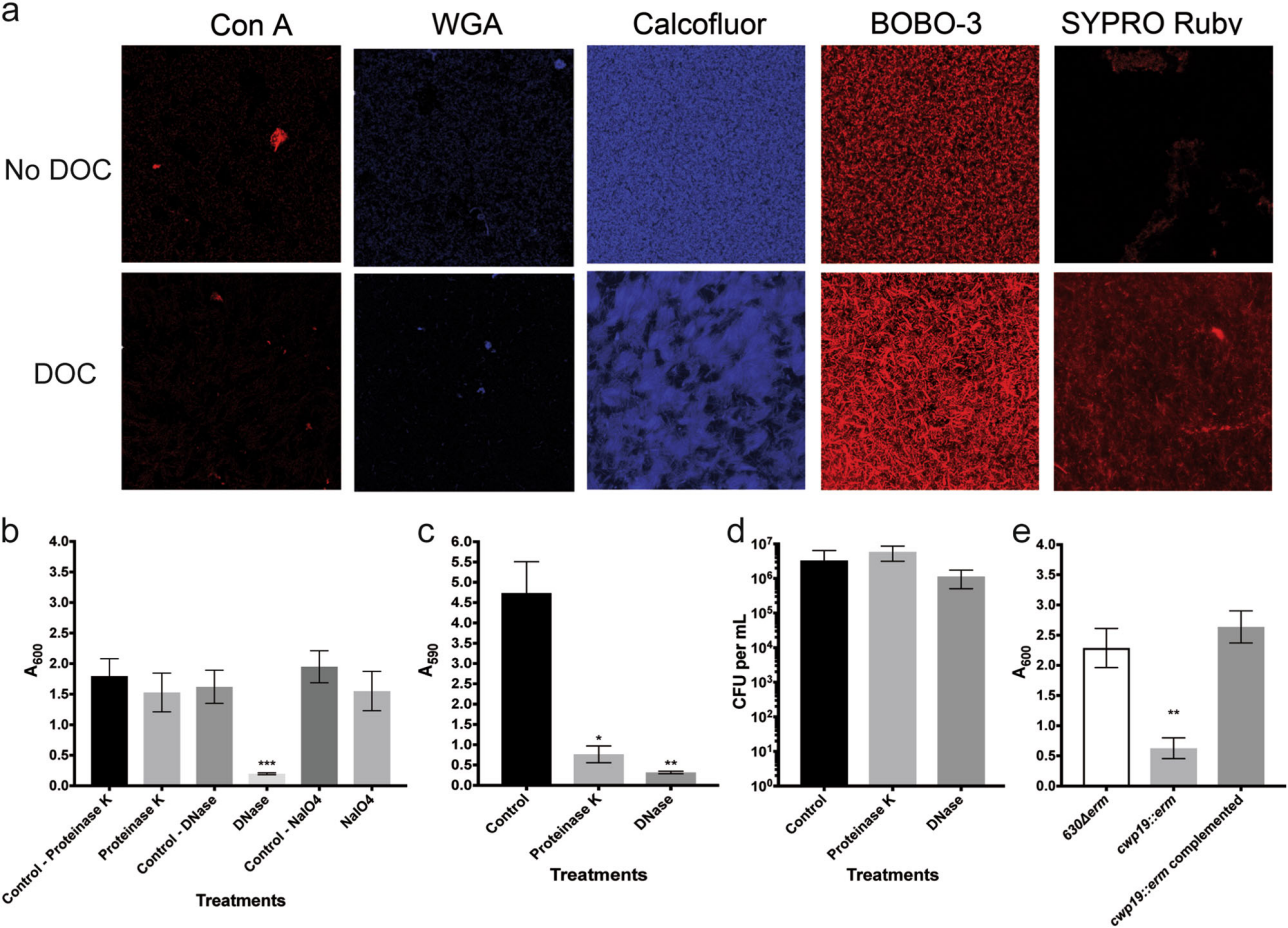
Analysis of the biofilm matrix composition in the absence and presence of DOC. **a** CLSM analysis of the biofilm stained with ConA-Cy3 (red/α-D-glucosyl or α-D-mannosyl residues), WGA-Cy3 (red/N-acetylglucosamine or sialic acid residues), calcofluor white (blue/β-1,3 or β-1,4 polysaccharides), BOBO-3 (red/eDNA), or Sypro Ruby (red/proteins). The CLSM observations were performed with unwashed biofilms. **b** Dispersion of 48 h biofilms with proteinase K, DNase I, and NaIO_4_. **c** Inhibition of biofilm formation by proteinase K and DNase I. **d** CFU of biofilms treated with proteinase K and DNase I. The viability assays were performed with unwashed biofilms. **e** Biofilm formation in BHISG with 240 µM DOC by the parental strain, the *cwp19* inactivated strain and *cwp19* complemented strain. Asterisks indicate statistical significance determined with a Kruskal–Wallis test followed by an uncorrected Dunn’s test (*p ≤ 0.05, **p ≤ 0.01 vs the control or 630∆*erm*.) Biofilm formation (**b**, **c**, **e**) was measured using a crystal violet assay that included two PBS washing before staining. Images are representative fields acquired from 3 different biological replicates. Each bar represents the mean of at least 5 biological replicates performed on different days. The error bars represent the standard error of the mean

During biofilm formation, bacteria produce a matrix generally made of extracellular DNA (eDNA), sugar polymers, and proteins that hold cells together. To characterize the matrix composition of the DOC-induced biofilm, we first compared the sugar content of cell-associated exopolysaccharides from *C. difficile* cells grown as biofilms with or without DOC. The quantity of hexose, galactosamine, and glucosamine was similar between both biofilm matrixes (Supplementary Fig. 6a). We also stained biofilms with dyes and lectins for residues of exopolysaccharides frequently found in biofilms. Concanavalin A (Con A) or wheat-germ agglutinin (WGA) did not stain either biofilms (Fig. 3a), suggesting that sugars containing α-D-glucosyl, α-D-mannosyl, N-acetylglucosamine, or sialic acid residues are below the level of detection or not accessible to the lectins. In contrast, the biofilms were stained with calcofluor white, which binds to β-1,3 or β-1,4 polysaccharides such as PSII,^30^ regardless of the growth conditions. The stain was mostly associated with the cells for either biofilm but did not appear in the space between aggregates (i.e., a biofilm structure; Fig. 3a). When we purified the matrix of the DOC-induced biofilms, we detected glycoproteins and high-molecular-weight fragments that were resistant to protease and DNase (Supplementary Fig. 6b). We also detected PSII using anti-PSII antibodies (Supplementary Fig. 6c). This indicates that PSII and glycoproteins are part of the biofilm matrix.

We then looked for eDNA and extracellular proteins by staining the biofilms with BOBO-3 and SYPRO Ruby Red, respectively.^31^ In the absence of DOC, no significant staining with SYPRO Ruby Red was observed and BOBO-3 did not stain between cells (Fig. 3a). In contrast, the staining pattern of the DOC-induced biofilm formed a net-like structure suggesting that the matrix of the DOC-induced biofilm is probably composed of eDNA and proteins. This was consistent with the presence of eDNA in the DOC-induced biofilm matrix as observed on an agarose gel (Supplementary Fig. 6d). In agreement with the eDNA staining, we observed that pre-established DOC-induced biofilms were rapidly dispersed when treated with DNase for 1 h (Fig. 3b). Interestingly, we observed that inactivation of the *cwp19* gene encoding the major autolysin involved in cell lysis when grown in BHI,^32^ failed to form a biofilm in BHISG with DOC (Fig. 3e). This supports the observation that accumulation of eDNA during cell lysis contributes to biofilm formation.

In contrast, the same treatment performed with proteinase K and NaIO_4_ (denaturing polysaccharides) did not significantly reduce the biomass of the DOC-induced biofilm (Fig. 3b). However, we observed that biofilm were dispersed without affecting bacterial viability when we incubated DOC-induced biofilms with proteinase K or DNase for 24 h (Fig. 3c, d). Taken together, our results indicate that eDNA, proteins, and exopolysaccharides such as PSII, are incorporated into the matrix during the maturation step but only eDNA is required for the stability of the DOC-induced biofilm while proteins and eDNA are needed for the assembly of the biofilm.

### Antimicrobial resistance of DOC-induced biofilms

Growth as a biofilm often reduces the sensitivity of bacteria to antimicrobial agents. Thus, we tested whether the DOC-induced biofilm can protect *C. difficile* against bactericidal concentration of DOC, antibiotics typically used for CDI therapy and human antimicrobial peptides produced in the large intestine. We first established the bactericidal concentration for DOC. Complete eradication of the planktonic cells was achieved at 1000 µM and only 500 CFU were detected at 600 µM (Table 1). We then tested if bacterial cells in the DOC-induced biofilm were more tolerant to bactericidal concentration of DOC. Total eradication of the biofilm was achieved at 2000 µM and, unlike planktonic cells, 5 × 10^4^ and 5 × 10^6^ CFU were detected at 1000 and 600 µM, respectively (Table 1). These results confirmed that pre-exposure to sub-inhibitory concentration of DOC decreases the sensitivity of *C. difficile* to bactericidal concentration of DOC.

**Table 1:** CFU remaining after exposure of planktonic cells and DOC-induced biofilm to increasing concentration of DOC

Cellular state	Concentration of DOC					
	0 µM	240 µM	480 µM	600 µM	1000 µM	2000 µM
DOC-induced biofilm	1 × 10^8^	5 × 10^7^	5 × 10^7^	5 × 10^6^	5 × 10^4^	<1 × 10^1^
Planktonic cells	5 × 10^7^	1 × 10^8^	5 × 10^6^	5 × 10^2^	<1 × 10^1^	<1 × 10^1^

We then determined the minimum inhibitory concentration (MIC) of vancomycin, metronidazole, and LL-37 for planktonic cells of strain 630∆*erm* grown in BHISG with or without DOC (Table 2). The MIC values of planktonic cells in BHISG were 2–16 times higher than the published data.^33,34^ The MIC values for vancomycin and metronidazole, in the presence of 240 µM DOC, were reduced by 32-fold whereas the MIC for LL-37 decreased only by 2-fold (Table 2). In addition to measuring MIC, we measured the minimal bactericidal concentration for biofilms (MBCB), which determines the concentration of antibiotic inhibiting regrowth or killing cells present in a biofilm and provides a more relevant view of the antibiotic resistance in biofilm.^35^ The MBCB for the DOC-induced biofilm (MBCB_DOC_) was determined for vancomycin, metronidazole, or LL-37. The MBCB_DOC_ was over 10,000- and 13,000-fold higher than the MIC_DOC_ for metronidazole and vancomycin, respectively or 4-fold higher for LL-37 than the MIC_DOC_ (Table 2). This confirms that DOC-induced biofilm cells of *C. difficile* have a lower sensitivity to antibiotic than planktonic cell grown in the presence of DOC.

**Table 2:** Susceptibility of *C. difficile* strain 630∆*erm* to vancomycin, metronidazole, and LL-37 in the presence of DOC or grown as biofilms

Antibiotics	Published MIC^a^ (μg/mL)	MIC (μg/mL)	MIC_DOC_ (μg/mL)	MBCB_DOC_ (μg/mL)
Vancomycin	1.0	1.5	0.047	625
Metronidazole	0.1	1.5	0.047	500
LL-37	16	31.25	16.125	62.5

^a^refs ^33,34^

### Repression of spores and toxins by DOC

**Fig. 4:**
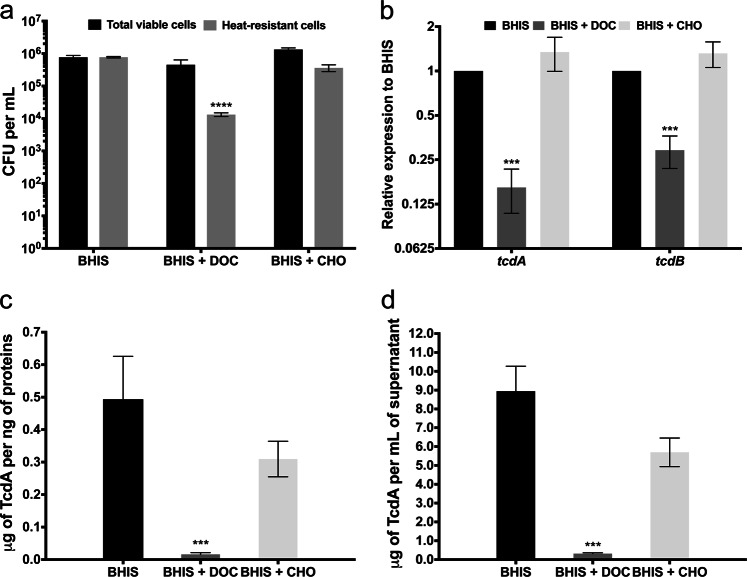
DOC represses both sporulation and toxin production of *C. difficile*. **a** CFU of viable cells (black) and heat-resistant spores (gray) in the absence or presence of 240 µM CHO or 240 µM DOC. The sporulation assays performed with unwashed biofilms. **b** Relative expression levels of *tcdA* and *tcdB* measured by qRT-PCR in cells grown in the absence or presence of 240 µM DOC or 240 µM CHO. Relative expression levels (∆∆Ct method) are the ratio of mRNA level in the presence of bile salts to the mRNA level in the absence of bile salts. Reactions of qRT-PCR were normalized using DNA *polIII* (*CD1305*), *rpoA* (*CD0098*), *pgi* (*CD3285*), and *tpi* (*CD3172*). **c** ELISA-based quantification of TcdA production by cells grown in the absence or presence of 240 µM DOC or 240 µM CHO. Concentrations were standardized to the amount of protein as measured by the Bradford method. **d** ELISA-based quantification of TcdA release into the supernatant by cells grown in the absence or presence of 240 µM DOC or 240 µM CHO. Asterisks indicate statistical significance determined with a two-way ANOVA followed by a Fisher LSD test (***p ≤ 0.001, ****p ≤ 0.0001 vs BHIS). Each bar represents the mean of at least 6 biological replicates performed on different days and error bars represent the standard error of the mean

In order to uncover the molecular mechanism involved in the DOC-induced biofilm, we performed a RNAseq analysis comparing the transcription profiles of biofilms obtained after 48 h of growth in BHISG with or without DOC (see Methods). The time point was selected based on biofilm formation kinetics (Fig. 1d). A total of 1466 genes and 86 non-coding RNAs (ncRNA) had a significant differential expression in the DOC-induced biofilm when compared to non-induced biofilm with a fold change ≥2 (Supplementary Data 1). Among these, 893 genes (56%) and 43 ncRNA (48%) were up-regulated while 573 genes (44%) and 46 ncRNA (52%) were down-regulated. The differentially regulated genes were classified according to the predicted functional class of their encoded proteins (Supplementary Fig. 7). Several classes were identified as important during the DOC-induced biofilm formation including genes involved in metabolic adaptation, stress resistance, cell envelope reorganization, and toxinogenesis (Supplementary Data 1). In contrast, we observe that few genes associated with sporulation are up-regulated, suggesting that the sporulation process is probably not initiated in the presence of DOC. In agreement, we found low levels of spores in the non-induced biofilm (1%) and fewer spores in the DOC-induced biofilm (0.1%). These low spore percentages might be due to the presence of glucose in the medium, a known repressor of sporulation.^36^ To better evaluate the effect of DOC on sporulation, *C. difficile* was grown in BHIS in the absence of glucose with or without 240 µM DOC (i.e., conditions that did not induce biofilm formation; Supplementary Fig. 4). After 7 days, we observed a reduction of 2 logs in the total number of spores in BHIS with DOC when compared to cells grown in BHIS or BHIS with CHO (Fig. 4a).

We also found that toxin-encoding genes were downregulated in the DOC-induced biofilm (Supplementary Data 1), a finding confirmed by qRT-PCR. However, the RNA samples used for the transcriptome were obtained from biofilms produced in BHIS containing glucose, which is also known to repress toxin synthesis.^36^ To determine the impact of DOC on toxin synthesis, we prepared RNA from cells grown for 48 h in BHIS and BHIS with DOC or CHO. The expression of *tcdA* and *tcdB* was down-regulated by 6- or 3-fold in BHIS with DOC when compared to BHIS or BHIS with CHO, respectively (Fig. 4b). We confirmed by ELISA that the addition of DOC to BHIS strongly reduced production and release of toxin A, while the addition of CHO had little or no effect (Fig. 4c, d). Taken together, these results indicate that toxin production and sporulation are repressed in DOC-induced biofilm by the combined effect of DOC and glucose.

### Biofilm formation is driven by metabolic and stress responses

**Fig. 5:**
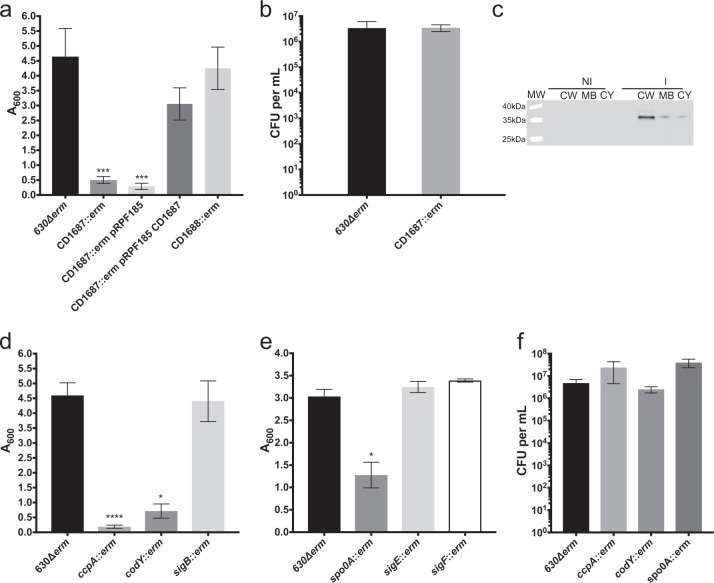
Biofilm formation in the presence of DOC requires the CD1687 and is regulated by CcpA, CodY, and Spo0A, but not by SigB. **a** Biofilm formation by the parental strain (630∆*erm*), *CD1687* mutants, a *CD1687* mutant with an empty vector (pRPF185) and a *CD1687* complemented strain (pRPF185::CD1687) grown in BHISG with 240 µM DOC. The inducer Atc (100 ng/mL) is added at the beginning of the experiment and every 24 h until the end of the experiment. **b** CFU of the parental strain (630∆*erm*) and the *CD1687* mutant. **c** Localization of the CD1687 lipoprotein in the presence (I) or absence (NI) of the inducer; cell wall fraction (CW); membrane fraction (MB), cytoplasm fraction (CY). Samples are derived from the same experiments and processed in parallel. **d** Biofilm production by the parental strain (630∆*erm*), *codY*, *ccpA*, and *sigB* mutants grown in BHISG with 240 µM DOC. **e** Biofilm production by the parental strain (630∆*erm*), *spo0A*, *sigE*, and *sigF* mutants grown in the BHISG with 240 µM DOC. **f** CFU by the parental strain (630∆*erm*), *codY*, *ccpA*, and *spo0A* mutants grown in the BHISG with 240 µM DOC. Asterisks indicate statistical significance determined with a Kruskal–Wallis test followed by an uncorrected Dunn’s test (*p ≤ 0.05, **p ≤ 0.01, ****p ≤ 0.0001 vs 630∆*erm*). Biofilm formation (**a**, **d**, **e**) was measured using a crystal violet assay that included two PBS washes before staining. The viability assays (**b**, **f**) were performed with unwashed biofilms. Each bar represents the mean and the error bars represent the standard error of the mean of at least 5 biological replicates performed on different days

The transcriptomic analysis showed that long-term exposure to DOC reorganizes metabolism of *C. difficile* (Supplementary Fig. 7 and Supplementary Data 1). Notably, genes involved in glycolysis, succinate and pyruvate metabolism, and the reductive acetyl-CoA pathway are down-regulated in the DOC-induced biofilm (Supplementary Data 1 and Supplementary Fig. 8). Furthermore, several genes encoding symporters, antiporters, and transporters involved in the uptake of sugars other than glucose are up-regulated. In addition, amino acid metabolism is modulated in DOC-exposed cells including those involved in Stickland reactions (Supplementary Fig. 8). On the other hand, genes encoding glycerol and glycerol-phosphodiester degradation, amino acids (proline) and fructose metabolisms as well as those encoding anhydromuropeptide recycling and peptidoglycan, lipid and fatty acid biosynthesis are up-regulated (Supplementary Data 1 and Supplementary Fig. 8). Finally, several stress-associated genes are differently regulated in biofilms in the presence of DOC (Supplementary Data 1). For example, genes described as part of the cold-shock response, often described in the protection of cells against stress such as osmotic shock and oxidative stress,^37^ are up-regulated. In addition, DOC probably induce a profound cell envelope reorganization due to the up-regulation of genes encoding cell wall proteins (CWPs), and putative enzymes associated with cell wall remodeling, peptidoglycan reticulation, peptidoglycan biosynthesis, and PSII biosynthesis (Supplementary Data 1). We selected from these up-regulated genes a predicted lipoprotein, CD1687 and tested its involvement during biofilm formation induced by DOC. This gene is located upstream of two genes (*CD1688*–*CD1689*) encoding a two component regulatory system (TCS) similar to the TCS CiaR-CiaH of *Streptococcus pneumoniae* TIGR4, known to play a role in biofilm formation.^38^ All three genes of the *CD1687*–*CD1689* operon were up-regulated 6–8-fold in the DOC-induced biofilm (Supplementary Data 1). We observed that DOC-induced biofilm formation by the *CD1687* mutant was greatly reduced (Fig. 5a) and complementation restored the biofilm phenotype (Fig. 5a). Moreover, the numbers of CFU for the parental and the *CD1687* mutant strains were similar (Fig. 5b) indicating that the phenotype is not due to a growth defect. As shown in Fig. 5c, CD1687 was mainly detected in the cell-wall fraction demonstrating that CD1687 is localized at the cell surface. To ensure that the effect of the *CD1687* deletion was not due to a polar effect on the TCS genes, *CD1688* was also inactivated and no effect on biofilm formation was observed (Fig. 5a). All together these results indicate that the previously uncharacterized CD1687 is a factor required for formation of a DOC-induced biofilm.

Overall, it appears that *C. difficile* is shifting its primary metabolism for the synthesis of cell wall and lipid membrane precursor and reroutes its metabolism toward the use of alternative source of carbon to generate energy as a response to DOC exposure. A predicted consequence of this metabolic shift is a modification of the cells surface promoting biofilm formation.

### Metabolic regulators are required for biofilm formation

We know that the global regulators CcpA and CodY and the master regulator of sporulation, Spo0A, are involved in metabolic shifts in response to the nutrient capacity of the surrounding environment^39,40^ and control biofilm formation in several species.^16,41,42^ In our transcriptome analysis, *codY*, *ccpA*, and *spo0A* are up-regulated (Supplementary Data 1) and their inactivation decreased biofilm formation in the presence of DOC (Fig. 5d, e) without affecting bacterial viability (Fig. 5f). In addition, the effect of *spo0A* inactivation on the DOC-induced biofilm is independent of sporulation and is probably due to its effect on metabolism, since the inactivation of *sigE* or *sigF* encoding sporulation-specific sigma factors has no effect on biofilm formation (Fig. 5e). Thus, CodY, CcpA, and Spo0A must control critical steps of biofilm formation in response to DOC, including metabolic responses. In *C. difficile*, the alternative sigma factor SigB is a key factor of the general stress response.^43^ However, inactivation of *sigB* did not have an effect on DOC-induced biofilm (Fig. 5d), indicating that SigB is not involved in this process. Based on the fact that more than 100 genes encoding regulators are up-regulated (Supplementary Data 1), exposure to DOC probably induces several regulatory responses rather than a general stress response

### *Clostridium scindens* induces *C. difficile* biofilm

**Fig. 6:**
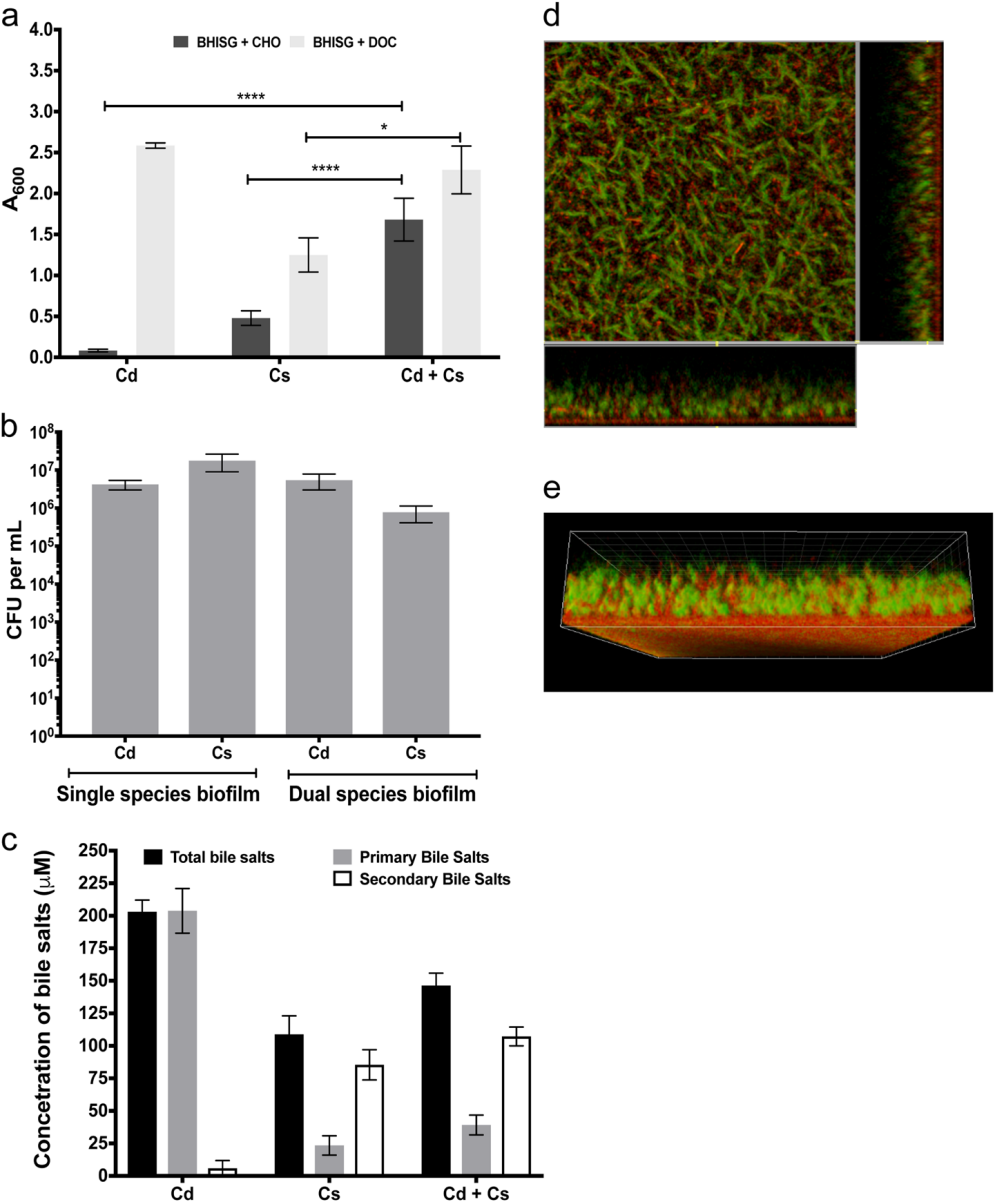
*C. difficile* biofilm formation enhanced by *C. scindens*. **a** Single-species and dual-species biofilm formation by *C. difficile* (Cd) and *C. scindens* (Cs) grown in the presence of 240 µM CHO or 240 µM DOC. Biofilm formation was measured using a crystal violet assay that included two PBS washing before staining. **b** CFU of *C. difficile* and *C. scindens* in single- and dual-species biofilms grown in the presence of 240 µM CHO in BHISG. The CFU counts were performed on unwashed biofilms. **c** Conversion of CHO to DOC by *C. difficile* and *C. scindens* in single- and dual-species cultures. **d** CLSM images and **e** 3D-reconstruction of a dual-species biofilm stained with Syto-61 (Red). Green cells are auto-fluorescent *C. difficile*. The CLSM analyses were performed with unwashed biofilms. Asterisks indicate statistical significance determined with a two-way ANOVA followed by a Fisher LSD test (****p ≤ 0.0001 vs CD + CS in BHISG + CHO). The error bars represent the standard error of the mean. Each bar represents the mean of at least 4 biological replicates performed on different days

To mimic the impact of DOC on *C. difficile* biofilm formation in the context of the gut environment, we used *Clostridium scindens* ATCC 35704 (Supplementary Table 2), a bile acid 7α-dehydroxylating bacterium that belongs to the limited number of species of the human intestinal bacteria able to convert CHO to DOC.^44,45^ Formation of mono- and dual-species biofilm by *C. difficile* and *C. scindens* was measured after 72 h of growth in BHISG with or without 240 µM CHO. In a mono-species culture, CHO does not induce biofilm formation of *C. difficile* but moderately induces biofilm formation of *C. scindens* (Fig. 6a). *C. scindens* biofilms were also produced in BHISG with DOC but was weaker than those produced by *C. difficile* (Fig. 6a). However, when *C. difficile* and *C. scindens* grew together, we observed a significant increase in biofilm formation in the presence of CHO and this biofilm was stronger than the *C. scindens* biofilms obtained in the presence of CHO or DOC (Fig. 6a). The CFU of the total population of both species were then counted to determine the proportion of *C. difficile* and *C. scindens* within dual-species biofilms in presence of CHO (Fig. 6b). In mono-species biofilm, the CFU counts were similar between both bacterial species, whereas in dual-species biofilm, there was 10-fold more *C. difficile* than *C. scindens*. This suggests that both species have cooperative interactions when grown in BHISG with CHO and this leads to an increase amount of biofilm. This increase could be explained by the generation of DOC by *C. scindens* resulting in the induction of *C. difficile* biofilm.

To confirm this hypothesis, we measured the total bile salts and primary bile salts present in the medium collected from the mono- and dual-species biofilm grown in the presence of CHO. As mono- and dual-species cultures, *C. scindens* converted 79% and 75% of CHO into secondary bile salts, respectively (Fig. 6c, Supplementary Table 3). In the case of *C. difficile* mono-species culture, CHO was not converted into secondary bile salts (Fig. 6c, Supplementary Table 3). Taken together, our results strongly support that in a dual-species culture, *C. scindens* induces biofilm formation of *C. difficile* by converting CHO into DOC.

To visualize the dual-species biofilms, we took advantage of the specific green autofluorescence of *C. difficile* and the fact that *C. scindens* is rapidly stained by SYTO-69 (<30 min) when compared to *C. difficile*, (Fig. 6d and Supplementary Fig. 9). The CLSM analysis confirmed that both species were present (Supplementary Fig. 9) and the length of *C. difficile* cells increased in dual-species biofilms as observed in mono-species biofilms in BHISG with DOC (Figs 2b and 6d). Interestingly, we observed that the base of the biofilm had a larger proportion of *C. scindens* (Fig. 6e), suggesting that in co-culture, *C. scindens* probably attaches first and provide a binding substrate for *C. difficile*. Altogether, these results confirm that DOC is an inducer of biofilm formation in *C. difficile* and suggests that the 7α-dehydroxylating bacterium of the intestinal commensal microbiota might favor biofilm formation by *C. difficile*.

## Discussion

In this study, we showed that *C. difficile* forms biofilm in the presence of DOC. In several pathogenic bacteria, induction of biofilm formation in the presence of bile salts is generally viewed as an adaptive response contributing to virulence and bacterial survival during colonic infection.^10–13^ In the presence of DOC, *C. difficle* induces specific adaptive responses, including biofilm formation. This is promoted by a reorganization of the cells envelope and the tight adherence of *C. difficile* cells to surfaces and other cells via eDNA. Our transcriptomic analysis showed that membrane and energy metabolism is affected in the DOC-induced biofilm. In support of this, inactivation of metabolic regulators (*ccpA*, *codY*, and *spo0A*) and an uncharacterized lipoprotein (CD1687) resulted in a significant decrease in biofilm formation. In other bacterial species, adaptation to bactericidal concentration of bile salts can be achieved by inducing physiological changes, which include membrane permeability and transporter.^7,8^ For example, *Bifidobacterium animalis* induces the expression of genes encoding an ATPase complex in the presence of bile salts^46^ and the increase in intracellular ATP helps maintain the proton gradient by pumping protons outside the bacterial cells. Additionally, *Bifidobacterium longum* enhances xylose utilization in the presence of bile salts and uses the pentose phosphate pathway (i.e., bifid shunt) to produce reducing equivalents and energy.^47^ In DOC-induced biofilm, *C. difficile* likely uses alternate pathways to produce energy and to transport nutrients to circumvent the effects of the DOC on membrane integrity. For instance, cells present in the DOC-induced biofilm probably generate pyruvate either from the sialic acid utilization^48^ and the D-ribose-5-phosphate via the synthesis of chorismate or from the utilization of fumarate produced during the synthesis of dGTP and dATP. The majority of the predicted genes for these pathways are up-regulated (see Supplementary Fig. 8). In agreement, several symporters, antiporters, and sugar transporters were up-regulated in the DOC-induced biofilm, suggesting that specific molecules and sugars other than glucose can be used to maintain proper homeostasis and energy metabolism.

Since fermentable sugars are required for maximum biofilm formation in response to DOC, additional factors regulated by these sugars must be also involved in the process. In *Staphylococcus aureus*, the global regulator of the carbon catabolic repression (CCR) system, CcpA, controls biofilm formation in the presence of glucose by positively regulating the synthesis of the polysaccharide intracellular adhesin (PIA) and the holin CidE involved in the release of eDNA.^49^ In *C. difficile*, inactivation of *ccpA* reduced biofilm formation in response to DOC (Fig. 5). Interestingly, the expression of *pilA* or *pilW* encoding type 4 pilins (T4P) was down-regulated in a *ccpA* mutant.^36^ Consistent with the up-regulation of *ccpA* in the DOC-induced biofilm, both *pilA* and *pilW* genes are up-regulated in our transcriptomic analysis, while the flagellum cluster is down-regulated (Supplementary Data 1). This indicates that T4P expression might be regulated by metabolism-associated regulators and may be involved in biofilm formation in the presence of DOC. In *Clostridium perfringens*, both T4P and CcpA are required for maximum biofilm formation.^50^

Biofilm matrix is generally composed of polysaccharides, proteins, DNA, surfactants, glycolipids, lipids, and cations.^14^ We showed that the matrix of the *C. difficile* biofilm induced by DOC is mainly composed of eDNA while proteins and polysaccharides are present (Fig. 3). In several pathogenic bacteria such as *L. monocytogenes*, *Enterococcus faecalis*, and *Pseudomonas aeruginosa*, eDNA is a major component required for the initial development phase of biofilm formation.^51,52^ Autolysis is considered to be one of the sources of eDNA within single species biofilms.^52^ We recently showed that Cwp19 is involved in autolysis of *C. difficile* in BHI when cells reach stationary phase^32^ and could be required for the release of the eDNA needed to build the DOC-induced biofilm. Indeed, biofilm formation in the presence of DOC was reduced in the absence of Cwp19 (Fig. 3e) because inactivation of cwp19 probably prevents accumulation of eDNA during cell growth. A similar role has been suggested for the autolysin AtlE of *Staphylococcus epidermidis* since an *atlE* mutant releases significantly less eDNA and produce less biofilm.^53^ It is therefore conceivable that a fraction of the population undergoes controlled lysis, which provides sufficient eDNA to build a biofilm for the remaining living population.

Recent work suggests that in a normal microflora, 7α-dehydroxylating gut bacteria produce secondary bile salts preventing the onset of CDI.^54^ In addition, production of antibacterial compounds by the 7α-dehydroxylating producer *C. scindens* has also been suggested as an element that helps prevent CDI but it has yet to be proven in vivo.^55^ Based on an older study, we know that DOC inhibits the growth of *C. difficile* in vitro.^5^ In agreement with these results, we find that high concentrations of DOC (600 µM and above, Fig. 1c) prevent biofilm formation by inhibiting the growth of *C. difficile* (Supplementary Fig. 2). In contrast, a sub-lethal concentration of DOC (240 µM) induces biofilm formation (Fig. 1c), represses toxin production (Fig. 4b–d) and makes *C. difficile* resistant to concentrations of DOC which are lethal without pre-exposition (Table 1). Sub-lethal concentrations of DOC may be encountered by *C. difficile* in the intestinal tract during the restoration of the normal microbiota following cessation of specific anti-CD antibiotic treatment. During this transitional phase, the vegetative cells could respond to the presence of DOC and induce a transition towards a state that favors persistence in hostile environments. This adaptation may include the formation of biofilm. During this transition, *C. difficile* cells could become non-toxigenic and less sensitive to DOC, allowing *C. difficile* to persist asymptomatically when the normal microbiota is restored. Recurrence of CDI may occur if *C. difficile* is able to persist within the gastrointestinal tract or if a patient is re-infected with a new *C. difficile* isolate. Persistence of *C. difficile* is mainly correlated with its ability to sporulate during infection or to resist antibiotic exposure.^40^ However, relapse could be associated with the persistence of *C. difficile* as vegetative cells including biofilms, which are recognized as a cause of chronic infections.^14^ This would be consistent with the asymptomatic carriage of *C. difficile*, now accepted as a reservoir for transmission. In agreement with this, we observed in our study that the production of an antibacterial compound or 7α-dehydroxylation of CHO by *C. scindens* does not prevent the growth of *C. difficile* and the conversion of CHO to DOC induced biofilm formation for both bacteria (Fig. 6). Therefore, the DOC produced by the intestinal bacterial community would induce biofilm formation while repressing toxin production and sporulation. Overall, this would prevent pathology of CDI and could explain how *C. difficile* persists in the gastrointestinal tract after restoration of the normal microbiota, thus increasing the risk of relapses. Work is currently in progress to understand the contribution of the DOC-induced biofilm to the relapse of the disease.

## Methods

### Bacterial strains and culture conditions

Bacterial strains and plasmids used in this study are listed in Supplementary Table 2. *Escherichia coli* strains were grown in LB broth containing ampicillin (100 μg/mL) or chloramphenicol (15 μg/mL) when necessary. *C. difficile* strains were grown anaerobically (5% H_2_, 5% CO_2_, 90% N_2_) in BHIS or BHISG (BHIS with 100 mM glucose), supplemented with cefoxitin (25 μg/mL), thiamphenicol (15 μg/mL), or erythromycin (2.5 μg/mL) when necessary. Additionally, 100 ng/mL of anhydrotetracycline (Atc) was used to induce the *P*_*tet*_ promoter of the pRPF185 vector derivatives in *C. difficile*.^56^

### Single and dual species biofilm assays

To generate single species biofilm, overnight cultures of *C. difficile* were diluted 1:100 into fresh BHISG, 1 mL per well was deposited in 24-well polystyrene tissue culture-treated plates (Costar, USA) and the plates were incubated at 37 °C in anaerobic environment for 24–96 h. Solutions of individual bile salts, bile acids, or bile salt mixtures were filter sterilized under anaerobic conditions and added to the pre-equilibrated medium (Supplementary Table 1; final concentration 240–2000 μΜ). For the dual species biofilm assays, overnight cultures of *C. difficile* and *C. scindens* were normalized to an OD_600__nm_ of 1.00 and an equal volume of each (10 µL) was added to prepared wells (final volume 1 mL; 24-well plate). Cultures were incubated for 72 h at 37 °C under anaerobic conditions. At the indicated time points, biofilm biomass was measured using established methods.^17^ Briefly, spent media was removed and wells were gently washed twice with phosphate-buffered saline (PBS). Biofilms were air dried and stained with crystal violet (CV; 0.2% w/v) for 10 min. CV was removed by inversion; wells were washed twice then air-dried. Dye bound to the biofilm biomass was solubilized by adding 1 mL of a 50:50 ethanol–acetone solution and the absorbance, corresponding to the biofilm biomass, was measured at a *λ*_600nm_ with a plate reader (Promega GloMax Explorer). When needed, the solubilized dye was diluted for the reading to remain in the linear range of the spectrophotometer. Sterile BHISG with bile salts was used as a negative control and a blank for the assays.

### Bacterial cells count and sporulation assays

The CFU counts were carried out on unwashed biofilms. The planktonic phase was removed and sedimented cells were suspended in 1 mL of sterile PBS and plated on BHIS to determine the number of CFU. After 2 or 7 days, *C. difficile* spores were measured as previously described.^52^ In co-biofilms experiments, *C. difficile* and *C. scindens* were differentiated based on the colony morphology on BHIS plates.

### Inhibition and dispersion of biofilms

To measure biofilm inhibition, biofilms were formed for 48 h as described above and washed twice with sterile PBS. Fresh BHISG containing 100 μg/mL of proteinase K or 1 U/mL of DNase I was added and biofilm were incubated for 24 h. Biofilms were then washed, stained, and quantified as described above. Dispersion of established biofilms was performed as described previously.^31^ Briefly, DNase I (25 μg), proteinase K (25 μg), or NaIO_4_ (for a final concentration of 40 mM) was added directly to 48 h-old biofilms. Wells were treated under anaerobic conditions at 37 °C for 1 h with DNase I and proteinase K or for 2 h with NaIO_4_. Biofilms were then washed, stained, and quantified as described above.

### Microscopy

For CLSM, 48 h biofilms were grown in 96-well plates (µclear, Greiner Bio-One). 200 µL of pre-conditioned medium was added to each well and the plates were incubated at 37 °C under anaerobic conditions. The unwashed biofilms were then directly stained in green with 20 μM of SYTO9 (Life Technologies) to detect bacterial cells. In addition one of the following red or blue dye was added to stain cells or matrix components, i.e., 20 μM of propidium iodide, 25 μM of FM4-64, Alexa Fluor 633 conjugate to Con A (100 μg/mL) or WGA (1 mg/mL), calcofluor-white (50 μg/mL), BOBO-3 (0.1 μM) or FilmTracer SYPRO Ruby. After 30–45 min of incubation, Z-stacks of horizontal plane images were acquired in 1 μm steps using CLSM (Leica TCS SP8, INRA MIMA2 microscopy platform or Ultrapole) with a 63× immersion lens (NA = 1.2). Three stacks of images were acquired randomly on three independent samples. Fluorophores were excited and emissions were captured as prescribed by the manufacturer. Simulated 3D fluorescence projections were generated using IMARIS 7.0 software (Bitplane, Zürich, Switzerland).

### RNA isolation and quantitative reverse-transcriptase PCR

One full 24-wells plate was used to produce one replicate for one condition. After 48 h, the supernatant was removed by inverting the plate. The unwashed biofilms from BHISG were recovered in 20 mL of PBS. In BHISG with 240 μM DOC, the biofilm formed was washed twice and resuspended in 20 mL of PBS. The recovered washed (BHISHG + DOC) and unwashed (BHISG) biofilms were then centrifuged and total RNA was extracted from cell pellets as previously described.^57^ cDNA synthesis and qRT-PCR were carried as described before^57^ using primers listed in Supplementary Table 2.

### Whole transcriptome sequencing and analysis

Transcriptomic analysis for each condition was performed using 3 independent RNA preparations. The RNA samples were first treated using Epicenter Bacterial Ribo-Zero kit to remove the rRNA content. Then depleted rRNA fraction was used to construct strand-specific single end cDNA libraries according to manufacturers’ instructions using Truseq Small Stranded Total RNA sample prep kit, Illumina. Libraries have been sequenced by Illumina HiSeq2000 sequencer. Cleaned sequences were aligned to the reannotated *C. difficile* strain 630^58^ for the mapping of the sequences using Bowtie (Version 2.1.0). DEseq2 (version 1.8.3) was used to perform normalization and differential analysis using BHISG values as a reference for reporting the expression data of BHISG with 240 µM of DOC. Genes considered differentially expressed if the fold changes was ≥log_2_ 1.5 and their adjusted *p*-value to ≤0.05.

### *C. difficile* mutants and complemented strains

Construction of the *CD1687* mutant is described in Supplemental Material (Supplemental Fig. S10) and primers designed to retarget the group-II intron of pMTL007 to the desired gene are reported in Supplementary Table 2. To complement the *CD1687* mutant and express the CD1687-His protein in *C. difficile*, the *CD1687* gene with its RBS was amplified by PCR using appropriate primers (Supplementary Table 2) and cloned into the *Xho*I and *BamH*I sites of pRPF185^56^ to generate plasmid pDIA7000 and pDIA7001, respectively. Both plasmids were then transferred by conjugation into the *CD1687* mutant, yielding strain CDIP1169 and CDIP1170, respectively.

### Cellular localization of CD1687

Strain CDIP1170 was grown in 10 mL of BHIS for 10 h in the absence or presence of the inducer Atc (200 ng/mL). Cells were harvested by centrifugation and the cell wall, membrane and cytoplasm of the cell obtained according to the published methods.^59^ Briefly, cultures of *C. difficile* were harvested by centrifugation and resuspended in phosphate/sucrose buffer (0.05 m HNa_2_PO_4_, pH 7.0, 0.5 m sucrose) to an *A*_600nm_ of 20. Purified catalytic domain of endolysin CD27L was added to the cell suspensions at 30 μg/mL, and samples were incubated at 37 °C for 1 h. Spheroplasts were recovered by centrifugation and the supernatant was kept and corresponds to the cell wall (CW) fraction. The spheroplasts were then resuspended in phosphate buffer (0.05 m HNa_2_PO_4_, pH 7.0) containing 0.12 μg/mL DNase I at an *A*_600nm_ of 20 and incubated at 37 °C for 45 min. Suspensions were harvested at 25,000 × *g* for 10 min at 4 °C to separate membrane and cytoplasm fractions. Membranes were finally resuspended in phosphate buffer at an *A*_600nm_ of 20. All fractions were analyzed by western immunoblotting methods using anti-His antibodies. Uncropped blot is provided in Supplementary Fig. 11a.

### ELISA-based measurement of TcdA

Total TcdA amount was quantified from cytosol and supernatants by enzyme-linked immunosorbent assay (ELISA). Briefly, 1.5 mL of culture was harvested by centrifugation for 4 min at 13,000 rpm. Supernatants were collected and bacterial pellets were frozen at −20 °C. The frozen bacteria were thawed and sonicated. Cytoplasmic fraction were obtained by centrifuging the lysates (3 min at 13,000 rpm). The supernatant and cytosol fractions were then analyzed by ELISA. A 96-well immuno-plate (Nunc Maxisorp) was coated with 2 µg/mL of anti-toxin A rabbit polyclonal antibody (Abcam, Inc.) overnight at 4 °C. The coated wells were washed and incubated with Superblock blocking buffer (Thermo Fisher Scientific) for 1 h. The wells were then washed and air-dried. Samples were added into the wells, and the plate was incubated at 37 °C for 90 min. After washings, 0.2 µg/mL of an anti-toxin A chicken horseradish peroxidase (HRP) antibody (LSBio) was added in each well and the plate was incubated for 1 h at 37 °C. The wells were washed and incubated with a TMB (3,3′,5,5′-tetramethylbenzidine) substrate solution (Thermo Fisher Scientific) for 15 min in the dark. The stop solution (H_2_SO_4_; 0.2 M) was added into each well and the absorbance of the reaction was read at 450 nm.

### Titration of bile salts

Bile salts were quantified by measuring the production of NADH generated during the oxidation of the hydroxyl groups of bile salts by hydroxysteroid dehydrogenases (HSDHs). The total bile salts and primary bile salts were quantified according to the hydroxysteroid dehydrogenases (HSDHs) method.^60^ Reactions were initiated by the addition of 3α-HSDH (Worthington) at a final concentration of 80 µg/mL and absorbance was measured at 340 nm until it stopped increasing. Total concentrations of bile salts were calculated by generating standard curves and solving the equation *A*_340_ = *k*(CA + DCA + CDCA), where *k* is the extinction coefficient of NADH and CA is cholate. Quantitation of bile salts with a hydroxyl group at the C7 position (i.e., primary bile salts) was performed in a similar manner, except that supernatant from cells overexpressing 7α-HSDH (strain JG73^61^) was used to initiate the reaction and the equation solved was *A*_340_ = *k*(CA + CDCA). This supernatant was prepared as follows: JG73 was grown to mid-log phase, at which time IPTG (1 mM) was added to induce during 2 h overproduction of 7α-HSDH and cells were pelleted and frozen at −20 °C. On the day of the assay, the pellet was resuspended in 0.1 M sodium phosphate, 1 mM EDTA, pH 7, sonicated, and centrifuged for 20 min at 6000 × *g*. The supernatant was immediately used in the HSDH assay.

### Antimicrobial peptides and antibiotic sensitivity assay

The MIC was determined by broth microdilution,^35^ using a 96-well plate containing twofold dilutions of desired antibiotic or antimicrobial peptide inoculated with overnight culture diluted 1:100. For the DOC killing assay, overnight culture were diluted 1:100 in BHISG, grown to late exponential phase, and diluted to 1 × 10^7^ in BHISG containing the desired concentration of DOC. After 24 h at 37 °C, CFU were determined by serial dilution and by plating on BHIS.

The minimum bactericidal concentration for biofilms (MBCB) was performed using established methods.^35^ Briefly, biofilms were prepared as described above. After 48 h, biofilms were washed twice with sterile PBS and fresh BHISG containing DOC and dilutions of antibiotics or antimicrobial peptide were added to each well. Plates were incubated for 24 h at 37 °C. Biofilms were then resuspended and bacteria were serially diluted in BHIS to 1:1000 in 96-well plates sealed with a plastic film to create an anaerobic environment. Growth kinetics was monitored for 24 h as described in Supplementary Fig. 3. To measure the bactericidal effect of DOC, 48 h biofilms were washed and exposed to BHISG containing various concentration of DOC for 24 h at 37 °C. CFU were determined by serially diluting the resuspended biofilm and by plating on BHIS. The MBCB was considered to be the lowest concentration resulting in no detectable growth after 24 h.

### Statistical analysis

All biofilm assays, genetic inactivation, or toxin production were analyzed using a Kruskal–Wallis test followed by an uncorrected Dunn’s test. Data for CFU counts, dual-species biofilm, spore formation, and toxin expression were compared and analyzed using a two-way ANOVA followed by a Fisher LSD test. Cell size and the HEPES treatment were compared and analyzed by a two-tail Mann–Whitney test.

### Reporting summary

Further information on research design is available in the Nature Research Reporting Summary linked to this article.

## Supplementary information

Supplementary Information

Data Set 1

Reporting Summary

## Data Availability

RNA-Seq data generated in this study are available in the NCBI-GEO with accession no. GSE103952. RNA-Seq coverage can be accessed at http://mmonot.eu/COV2HTML/visualisation.php?str_id=-350001. Other data that support the findings of this study are available from the corresponding author upon reasonable request.

## Supplementary information

**Supplementary information** accompanies the paper on the *npj Biofilms and Microbiomes* website (10.1038/s41522-019-0087-4).
