# Supplementary material for: A microbiota-generated bile salt induces biofilm formation in Clostridium difficile
Source: NPJ Biofilms Microbiomes. 2019 May 9;5:14. doi: 10.1038/s41522-019-0087-4 (PMC6509328; doi:10.1038/s41522-019-0087-4)
Supplement: Supplementary file 1 — Supplementary Information [file 41522_2019_87_MOESM1_ESM.pdf]

Supplementary Methods 1

Analysis of the Biofilm matrix

To measure total hexose (Hex), galactosamine (GalN) and glucosamine (GlcN), biofilms were prepared as described in the Materials and Methods section. After 48h, the plate was inverted to remove the growth medium and the unwashed biofilms from 24 wells were resuspended in 2 mL of PBS. The suspension was passed through a 0.45 mm syringe needle and then ten times through a 0.30 mm syringe needle. Samples were centrifuged (2 700 × g for 10 min) to separate bacterial cells from the biofilm matrix and supernatants were filtered (22 μm) to remove residual cells. Samples were then precipitated in ethanol overnight at 4°C and the pellets were suspended in water. Carbohydrate content was analyzed using standard methods.

To harvest the biofilm matrix, biofilms were grown as described in the Materials and Methods section, washed twice with PBS and resuspended in 1.5 M NaCl (12 wells/mL). The biofilm suspension was then centrifuged (8 000 × g for 10 min) and the supernatant was collected and stored at -20°C. A volume (500 μL) was then treated with DNase (25 μg) and Proteinase K (25 μg) for 1h at 37°C. Samples were then analyzed by agarose gele eletrphoresis, SDSD-PAGE or immunodetection.

Immunodetection of PS-II

The samples were serially diluted and 5 μL were spotted on a PVDF membrane. The membrane was then blocked with 5% skim milk in Tris-buffered saline with Tween 20 (TTBS) for 1 h followed by an incubation with a rabbit anti-PSII antisera (1:8000; kindly supplied by Dr. Gayatri Vedantam, University of Arizona) in TTBS containing 5% (wt/vol) HRP-conjugated antibody (1:10,000) in TTBS containing 5% (wt/vol) skim

milk for 1h and developed with SuperSignal West Femto chemiluminescent substrate (ThermoFisher).

Construction of *CD1687* and *CD1688* mutant strain. The ClosTron gene-knockout system (1) was used to inactivate *CD1687* or *CD1688*. Primers were designed to retarget the group II intron of pMTL007 to the target gene (Supplementary Table 2) and were used to generate a 353-bp DNA fragment by overlap PCR according to the manufacturer's instructions. These PCR products were cloned into the *Hind*III and *BsrG*I restriction sites of the pMTL007 and were verified by DNA sequencing using the pMTL007-F and pMTL007-R primers (Supplementary Table 2). The derived pMTL007 plasmids were transformed into *E. coli* strain HB101 (RP4) and transferred by conjugation into the *C. difficile* strain 630Δ*erm*. *C. difficile* transconjugants were selected by sub-culture on BHI agar containing thiamphenicol (15 µg/ml), and the integration of the group-II intron RNA into genes was induced and selected by plating onto BHI agar containing erythromycin (2.5 µg/ml). The chromosomal DNA of the transconjugants was extracted using the InstaGene Kit (BioRad), and PCR using the primers ErmRAM-F and ErmRAM-R (Supplementary Table 2) was used to confirm the erythromycin-resistant phenotype due to the splicing of the group-I intron from the group-II intron following integration. To verify the insertion of the group-II intron into target genes, PCRs with primers flanking the 5' ends of genes (Supplementary Table 2) and EBSu primer were performed to verify the integration of the group-II intron.

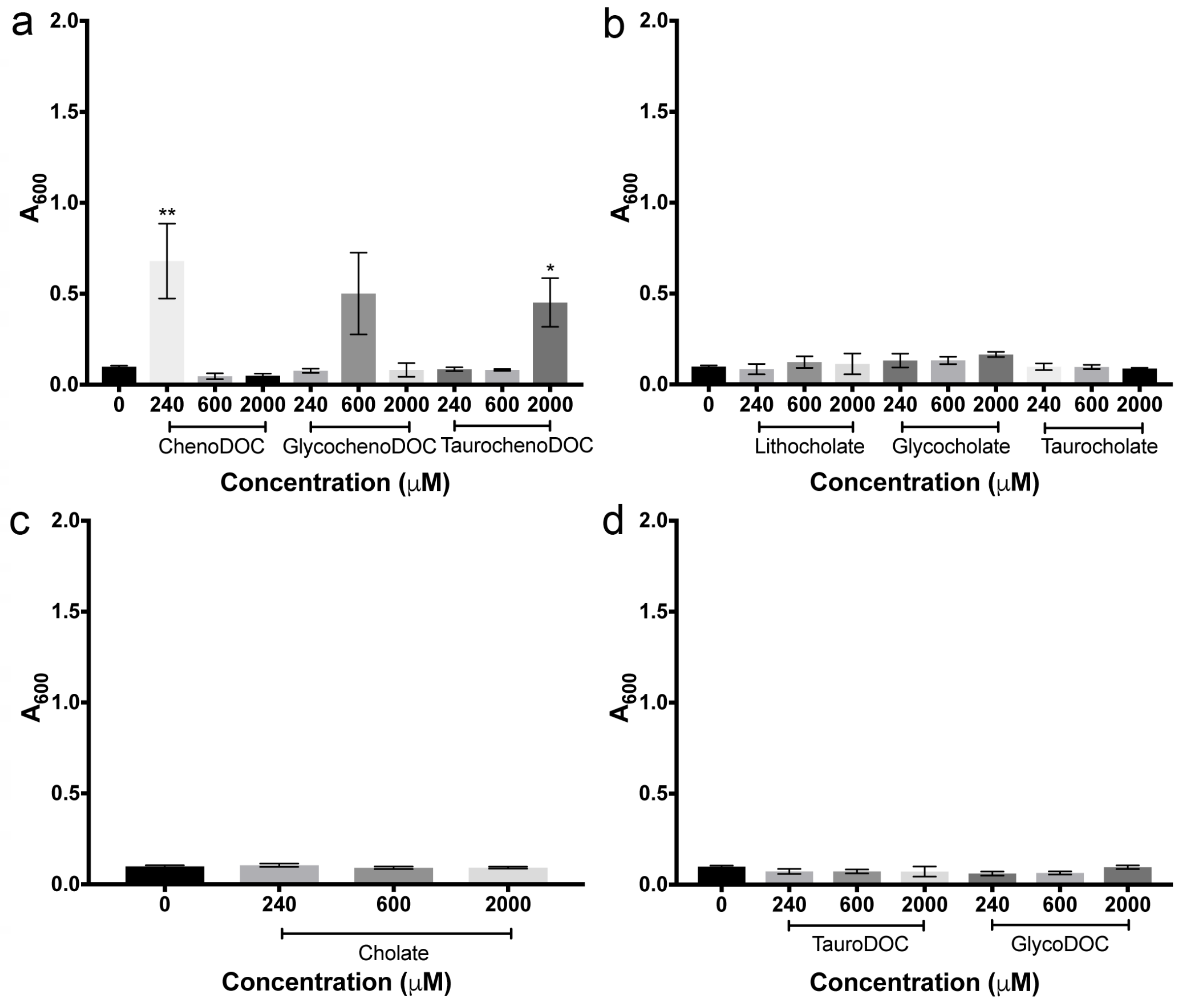

Supplementary Figure 1: Effect of chenodeoxycholate (ChenoDoc), glycochenodeoxycholate (GlycochenoDoc), taurochenodeoxycholate (TaurochenoDOC), lithocholate, glycocholate, taurocholate, cholate and taurodeoxycholate (TauroDOC), glycodeoxycholate (GlycoDOC) on biofilm formation. *C. difficile* was grown in BHISG in the presence of the indicated bile salt A: ChenoDOC, GlycoDOC, TauroDOC; B: lithocholate, glycocholate, taurocholate; C: cholate; D: TauroDOC, GlycoDOC. Biofilm formation was quantified at 72 h by staining with crystal violet. The crystal violet assay included two PBS washing before staining (see the Materials and Methods section). Asterisks indicate statistical significance determined by a Kruskal-Wallis test followed by an uncorrected Dunn's test (* p≤0.05, ** p≤0.001 ***

$p \leq 0.001$ vs BHISG with 0 mg/mL bile salts or 0 µM DOC). The error bars represent the standard error of the mean. Each bar represents the mean of at least 5 biological replicates performed on different days.

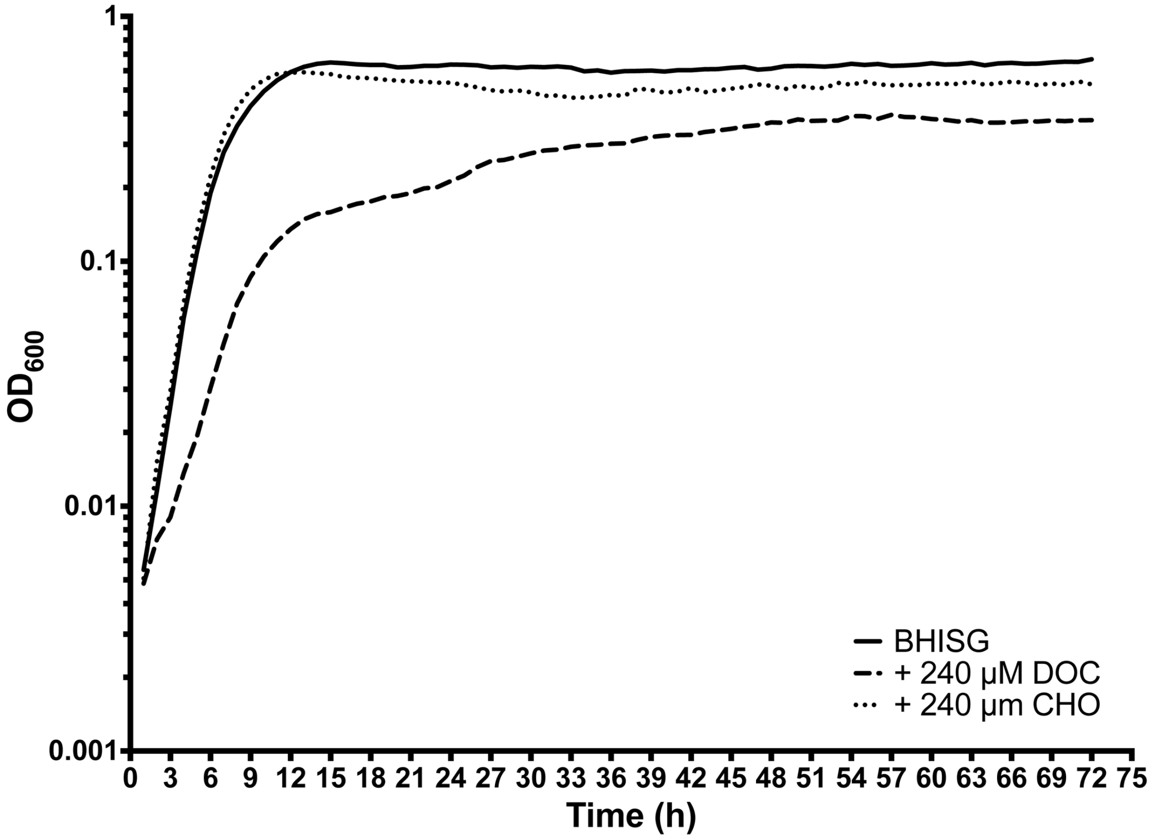

Supplementary Figure 2: Growth kinetics of *C. difficile* in BHISG with or without 240 µM CHO or 240 µM DOC. An overnight culture was diluted 1:100 in pre-equilibrated BHISG, BHISG with 240 µM DOC or BHISG with 240 µM CHO. These dilutions (1 mL) were transferred to a 24-well plate and the plate was sealed with a plastic film to create anaerobic environment. Growth kinetics was monitored using a plate reader (Promega GloMax Explorer) at 37°C with 10 s of shaking every 15 min and reading the OD_{600} every 1 h for 72 h. Error bars are omitted; each time point represents the average of 4 independent biological replicates.

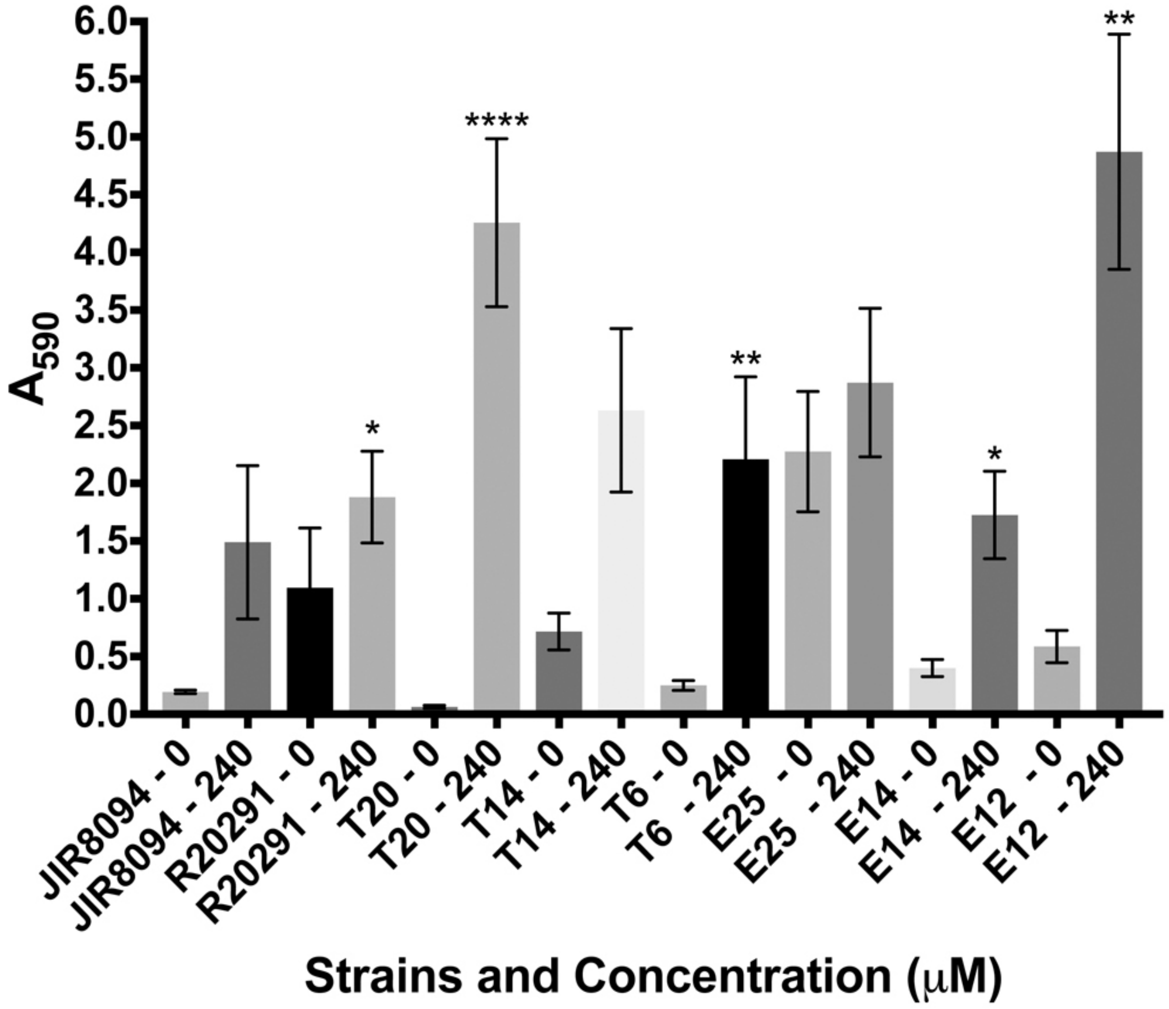

Supplementary Figure 3: Effect of DOC on biofilm formation of the R20291 strain and clinical isolates. *C. difficile* isolates (Supplementary Table 3) were grown in BHISG in the absence or presence of 240 μM DOC and biofilm formation was quantified at 72 h by staining with CV. The crystal violet assay included two PBS washing before staining. Asterisks indicate statistical significance determined by a Kruskal-Wallis test followed by an uncorrected Dunn's test (* $p \leq 0.05$ ** $p \leq 0.01$ **** $p \leq 0.0001$ vs 0 μM) for each respective isolates. The error bars represent the standard error. Each bar represents the mean of at least 3 biological replicates performed on different days.

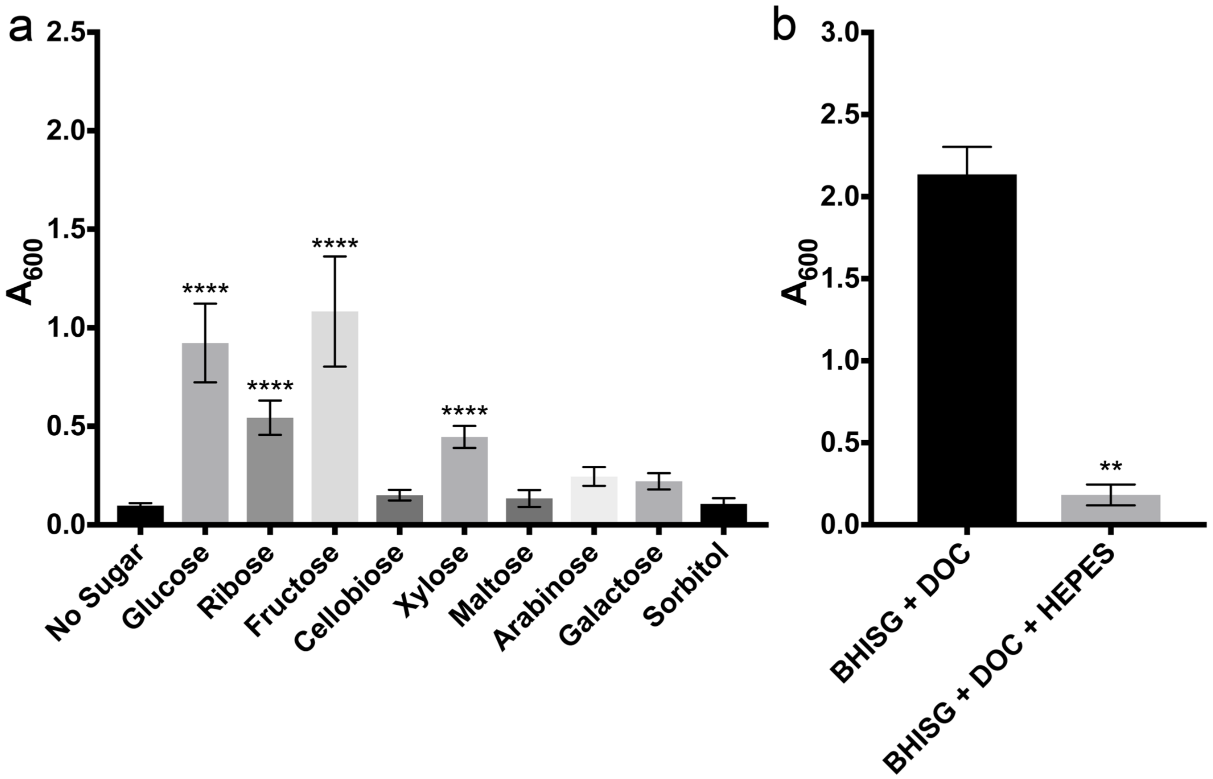

Supplementary Figure 4: Effect of PTS sugars and buffering on biofilm formation in the presence of DOC. Bacteria were grown in BHIS with 240 µM DOC and 50 mM of the specified sugar (A) or with 50 mM HEPES and 100 mM glucose (B). Biofilm formation was evaluated by staining with crystal violet after 72h (A) or 48h (B). The crystal violet assay included two PBS washing before staining. In panel A, asterisks indicate statistical significance determined with a Kruskal-Wallis test followed by an uncorrected Dunn's test (**** $p \leq 0.0001$ vs no sugar) and in panel B, asterisks indicate statistical significance determined with a two-tail Mann-Whitney test (** $p \leq 0.01$ vs no HEPES). The error bars represent the standard error of the mean. Each bar represents the mean of at least 6 biological replicates performed on different days.

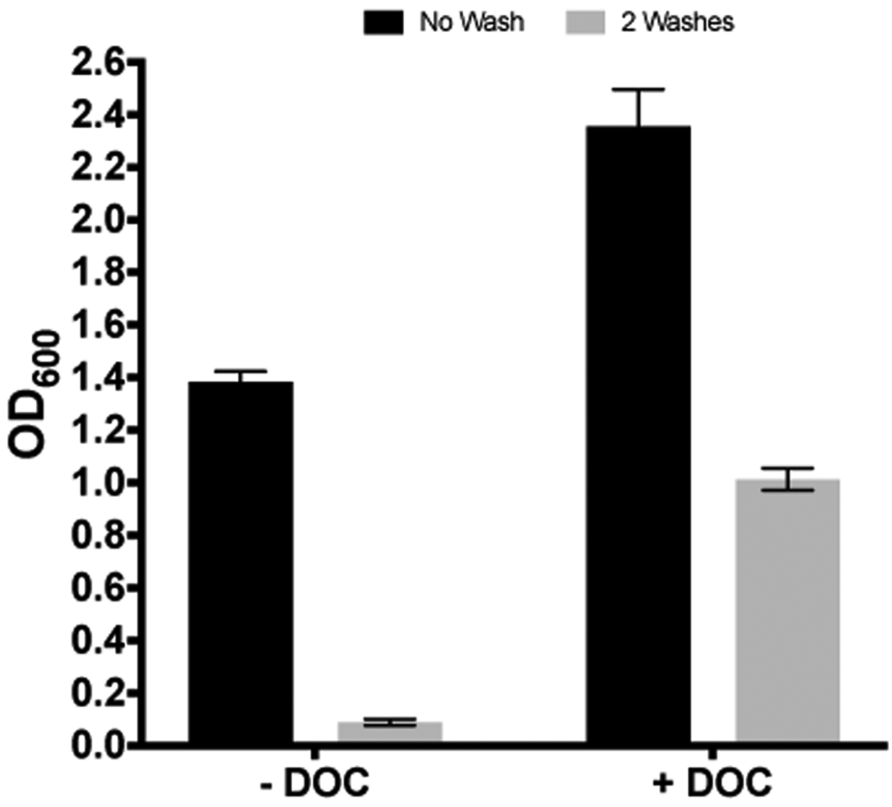

Supplementary Figure 5: Detachment of *C. difficile* cells from polystyrene by washing with PBS. The effect of washing on *C. difficile* biofilms was measured as follows: 48 h biofilms grown in BHISG without or with DOC were prepared as in the Material and Methods. The washed biofilms were then suspended in sterile PBS and the OD_{600} was measured with a spectrophotometer (Eppendorf Biophotometer). The error bars represent the standard error of the mean. Each bar represents the mean of 3 biological replicates performed on different days.

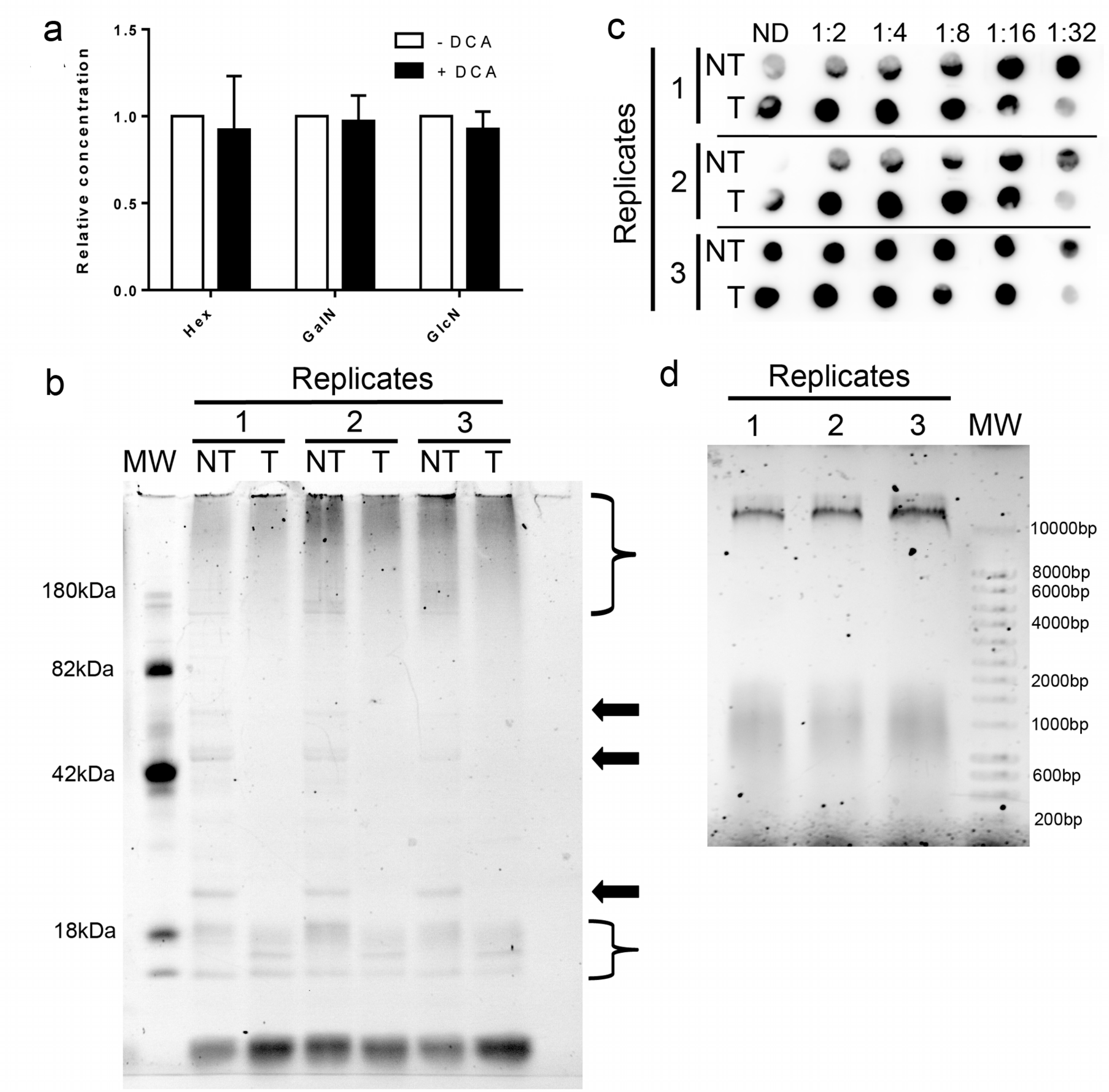

Supplementary Figure 6: Analysis of the composition of the biofilm matrix. (A) Analysis of the total hexose (Hex), galactosamine (GalN) and glucosamine (GlcN) content of unwashed biofilms. (B) SDS-PAGE analysis of the matrix isolated from 48h-biofilm grown in the presence of DOC and stained with the Pro-Q Emeral 300 glycoprotein stain kit (ThermoFischer). Arrows indicate glyproteins and curly brackets indicate the DNase and proteinase resistant matrix component. MW: CandyCane Glycoprotein Molecular Weight; NT: not treated; T: treated with DNase and Proteinase K. (C) Immunodetection of PS-II in the matrix of 48h-biofilm in the presence of DOC. NT: not treated; T: treated

with DNase and Proteinase K. (D) Agarose gel electrophoresis analysis of the matrix of 48h-biofilm of grown in the presence of DOC. MW: SmartLadder Molecular Weight. The error bars represent the standard error of the mean. Replicate represent independent biological replicates performed on different days.

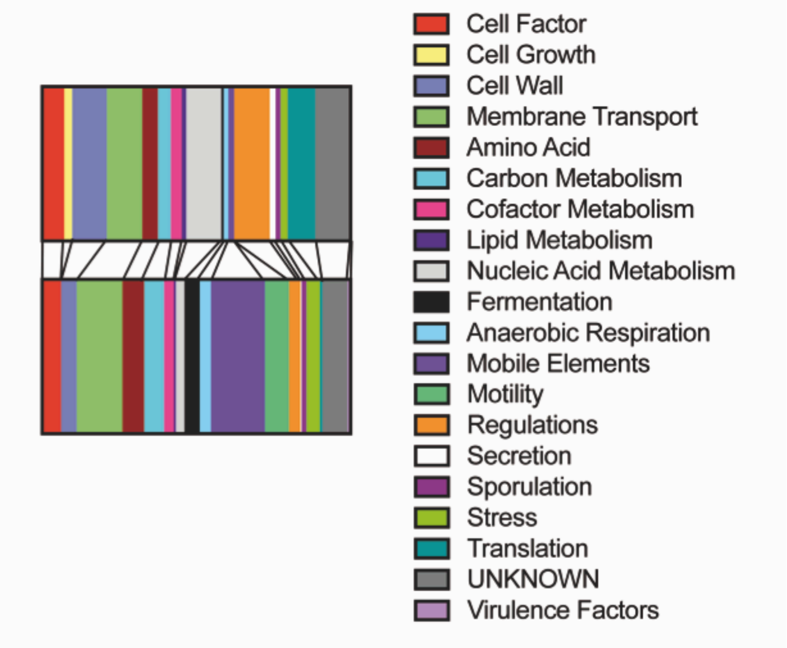

Supplementary Figure 7: A Graphical representation of the predicted function of differentially regulated genes.

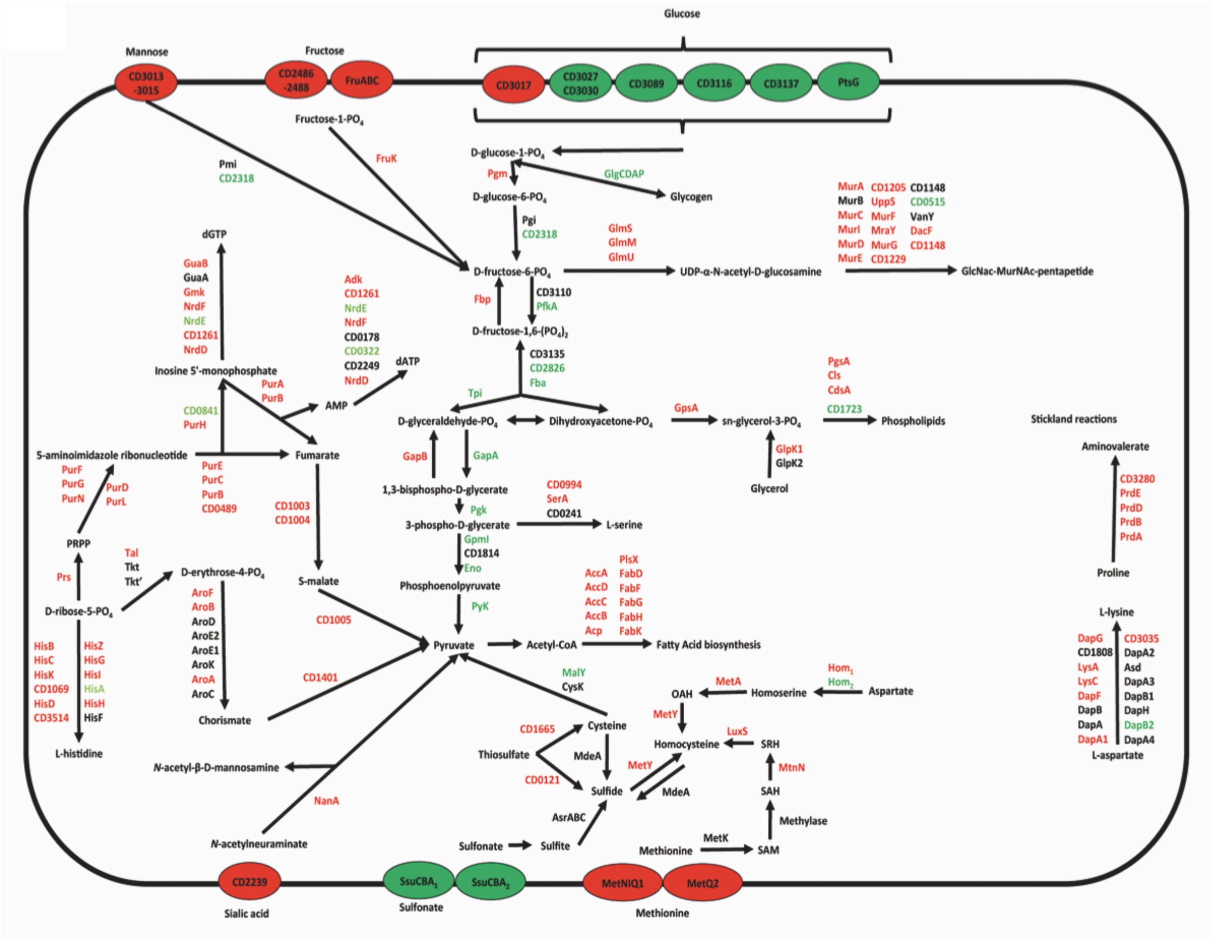

Supplementary Figure 8: Schematic representation of the metabolic pathways that are differentially regulated. Genes differentially regulated were analysed using the "omic" viewer option for the "Metabolism: Cellular Overview" function found in the BioCyc collection at biocyc.org to identify metabolic pathways involved

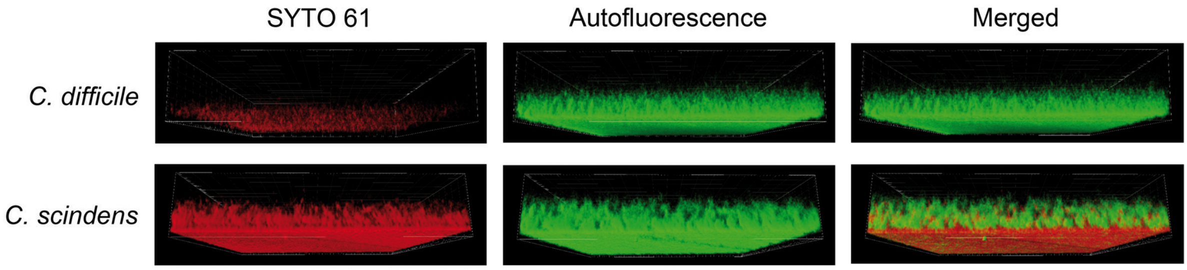

Supplementary Figure 9: CLSM analysis of *C. difficile* single and dual species biofilm with *C. scindens* in presence of CHO.

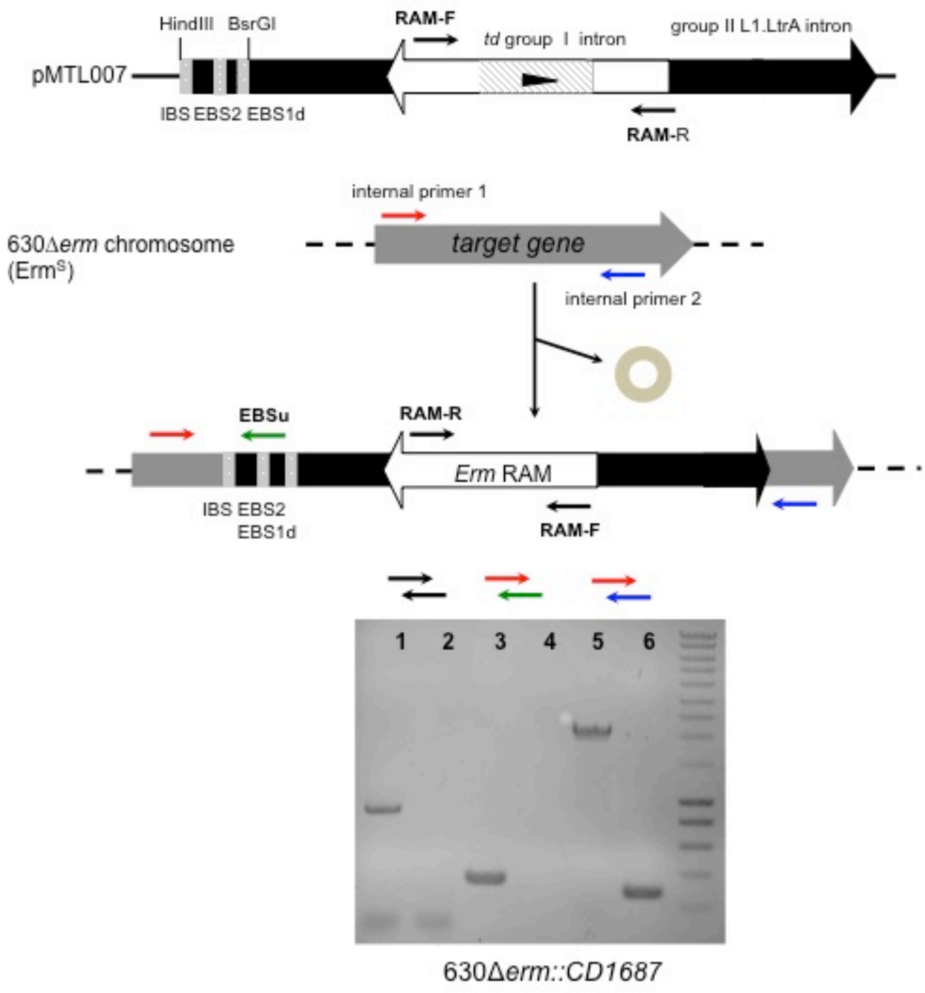

Supplementary Figure 10: Construction of *CD1687* mutant strain. Construction design to inactivate *CD1687* and the agarose gel confirming the insertion of the intron in *CD1687*.

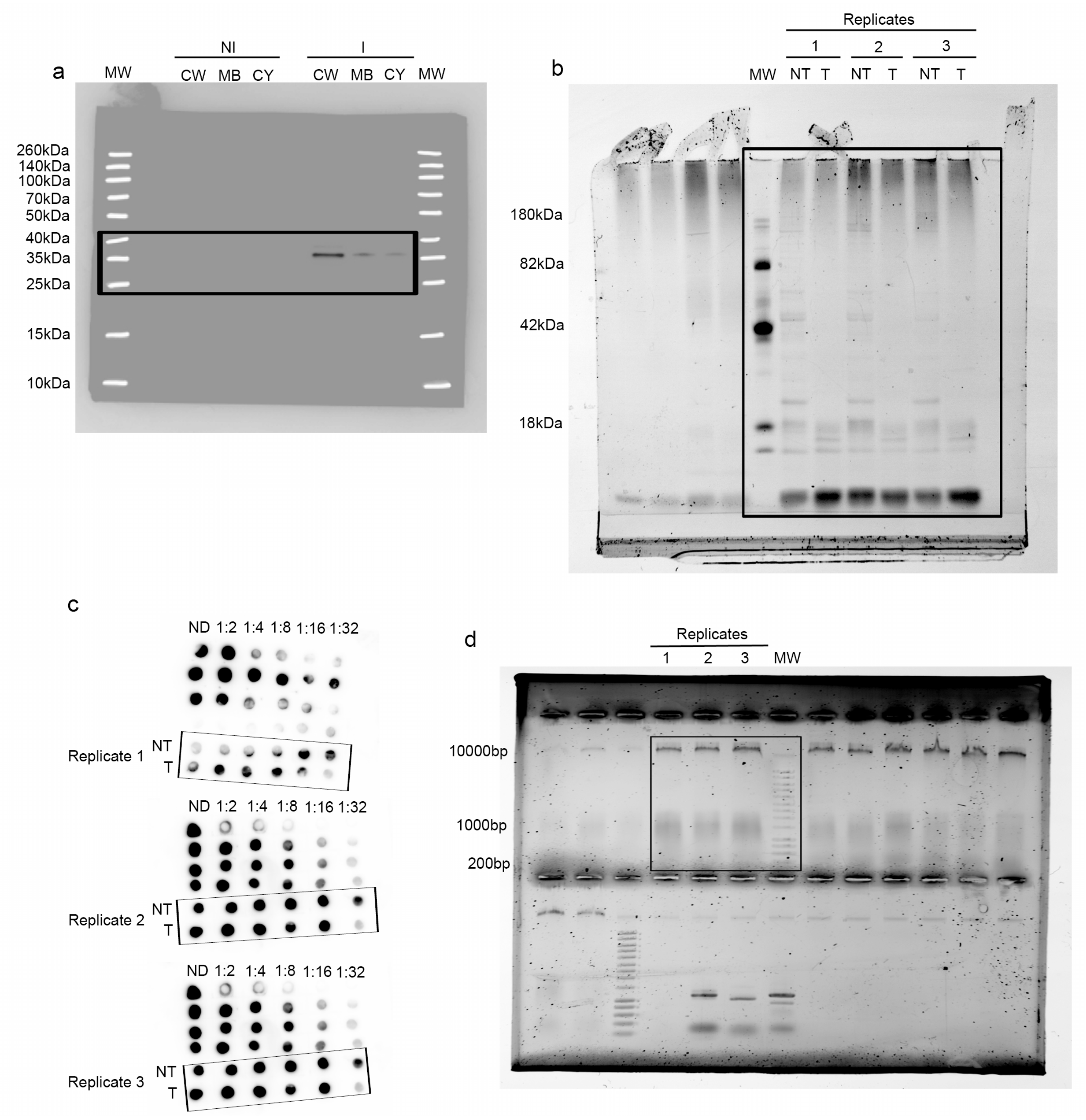

Supplementary Figure 11: Uncropped western immunoblot, SDS-page gel, dot blot and agarose gels. (a) Uncropped western immunoblot showing molecular weight marker from Figure 5. (b) Uncropped SDS-page gel stained with Pro-Q Emeral 300 glycoprotein stain from Supplementary Figure 6b. (c) Uncropped dot blot for the immunodetection of PS-II from Supplementary Figure 6c. (d) Uncropped agarose gel showing molecular weight marker from Supplementary Figure 6d. Boxes indicate the cropped area for the figures.

Table S1: List of bile acids and bile salts and their respective catalogue number and diluent.

Bile acid or bile salts	CAS number	Sigma Catalogue Number	Diluent
Bile salt extract		B3301	Water
Sodium cholate	206986-87-0	C1254	Water
Cholic acid	81-25-4	C1129	Glacial acetic acid
Sodium Chenodeoxycholate	2646-38-0	C8261	Water
Chenodeoxycholic acid	474-25-9	C9377	Ethanol
Sodium deoxycholate	302-95-4	D6750	Water
Deoxycholic acid	83-44-3	D2510	Ethanol
Lithocholic acid	434-13-9	L6250	Ethanol
Sodium taurodeoxycholate	6009-98-9	T6260	Water
Sodium glycodeoxycholate	16409-34-0	G3258	Water
Sodium glycochenodeoxycholate	16564-43-5	G0759	Water
Sodium taurochenodeoxycholate	6009-98-9	T6260	Water
Sodium glycocholate	338950-81-5	G7132	Water
Sodium taurocholate	345909-26-4	T4009	Water

Table S2: List of strains, plasmids and primers used for qPCR and gene construct

Strains and plasmids	PCR-ribotype/Genotype	Resistance[1]	Reference
Clostridium scindens	ATCC 35704		2
630Δ*erm*	012	Tc	3
JIR8094	012	Tc	4
R20291	027	Em, Mox	5
T6	014	Cm, Tc	6
T14	106	Mox, Cm	6
T20	078	Cm, Tc	6
E12	106	Mox, Em	6
E14	014		6
E25	005	Cm	6
CDIP1168	630Δ*erm CD1687::erm*	Em	This study
CDIP1169	CDIP1168 pDIA6693	Cm, Tm	This study
CDIP1170	CDIP1168 pDIA6694	Cm, Tm	This study
CDIP1436	630Δ*erm CD1688::erm*	Em	This study
CDIP229	630Δ*ermsigB::erm*	Em	7
CDIP240	JIR8094 *codY::erm*	Em	L. Bouillaut
CDIP222	JIR8094 *ccpA::erm*	Em	8
CDIP3	630 Δ*erm spo0A*::erm	Em	9
AHCD532	630Δ*erm sigE*::*erm*	Em	10
AHCD533	630Δ*erm sigF*::*erm*	Em	10
CDIP636	630Δ*erm cwp19*::*erm*	Em	11
CDIP637	630Δ*erm cwp19*::*erm* pCWP19	Em	11
**Plasmids**			
pMTL007::Cdi-*CD1687* 86a	pMTL007	Cm, Tm	This study
pMTL007::Cdi-*CD1688*	pMTL007	Cm, Tm	This study
pDIA6993	pRPF185 CD1687	Cm, Tm	This study
pDIA6994	pRPF185 CD1687-His	Cm, Tm	This study
**Primer Name**	**Sequence**	**Restriction site**	**Primers Description**
QRTpolIII-F_Cdiff	TCCATCTATTGCAGGGTGGT		DNA *polIII* qPCR
QRTpolIII-R_Cdiff	CCCAACTCTTCGCTAAGCAC		DNA *polIII* qPCR
QRTBD107-rpoA	TATGGAGATATTTGTAGATAAAGGTAG AGGTT		*rpoA* qPCR
QRTBD108-rpoA	CCACAGGTAAAACACCTATTGGAA		*rpoA* qPCR
QRTBD43-pgi	CAGATGATGTAGGTGGTCGTTTTT		*pgi* qPCR
QRTBD44-pgi	AGCAGCAATAGGAAGTAACCCAAC		*pgi* qPCR

QRTBD001-tpi	TGAAGTTAGAATACAATACGGTGGA		*tpi* qPCR
QRTBD002-tpi	CTATATCACTTTGACCCATTATTTCG		*tpi* qPCR
QRTBD013-tcdA	TAATAAAAATACTGCCCTCGACAAA		*tcdA* qPRC
QRTBD014-tcdA	ATAAATTGCATGTTGCTTCATAACT		*tcdA* qPCR
QRTBD005-tcdB	CTGGAGAATGGAAGGTGGTT		*tcdB* qPCR
QRTBD006-tcdB	TTGATGGTGCTGAAAAGAAGTG		*tcdB* qPCR
CD1687-86a-IBS	AAAAAAGCTTATAATTATCCTTAGTTC ACCTTTTTGTGCGCCCAGATAGGGTG		CD1687 interruption
CD1687-86a-EBS1d	CAGATTGTACAAATGTGGTGATAACAG ATAAGTCCTTTTTCATAACTTACCTTTC TTTGT		CD1687 interruption
CD1687-86a-EBS2	TGAACGCAAGTTTCTAATTTCGATTTG AACTCGATAGAGGAAAGTGTCT		CD1687 interruption
1687verifF	CTTACACAGGCATTGAAAGAATTTATG		CD1687-Clostron verification
1687verifR	CCTTCATCTATATTTATAATTTCTGAT TTTCC		CD1687-Clostron verification
1688-223s-IBS	AAAAAAGCTTATAATTATCCTTAGATA CCCCTGTAGTGCGCCCAGATAGGGTG		CD1688 interruption
1688-223s-EBS1d	CAGATTGTACAAATGTGGTGATAACAG ATAAGTCCCTGTACTTAACTTACCTTTC TTTGT		CD1688 interruption
1688-223s-EBS2	TGAACGCAAGTTTCTAATTTCGATTGT ATCTCGATAGAGGAAAGTGTCT		CD1688 interruption
1688verifF	AGAAGAACTTAGCAAGCATTTTG		CD1688-Clostron verification
1688verifR	CTAGTTTCCTTCATTGCAGTAGC		CD1688-Clostron verification
CD1687-comp1	CCG**CTCGAG**CTACTATAAAATACTACT ATATGAGGTGCT	*Xho* I	CD1687-Clostron complementation
CD1687-comp2	CGC**GGATCC**TACCTTTATTTGTCTATC TTAACTT	*Bam* HI	CD1687-Clostron complementation
CD1687Histag	CGC**GGATCC**TACCTTCAGTGATGGTGA TGGTGATGTTTGTCTATCTTAACTTTA TGAGATTTTAAGTC	*Bam* HI	CD1687-His
pMTL007-F	TTAAGGAGGTGTATTTCATATGACCAT GATTACG		pMTL007 cloning verification
pMTL007-R	AGGGTATCCCCAGTTAGTGTTAAGTCT TGG		pMTL007 cloning verification
ErmRAM-F	ACGCGTTATATTGATAAAAATAATAAT AGTGGG		Verification of the erm insertion
ErmRAM-R	ACGCGTGCGACTCATAGAATTATTTCC TCCCG		Verification of the erm insertion
EBSu	CGAAATTAGAAACTTGCGTTCAGTAAA C		Verification of the group-II intron

Cm : chloramphenicol; *Tm* : Thiamphenicol; *Em* : Erythromycin; Mox: Moxiflaxine; Tc: tetracycline

Table S3: Bile salt concentration in the culture supernatant of *C. difficile*, *C. scindens* and in co-culture

Strains and Replicates	Total bile salts	Primary bile salts	Secondary bile salts
C. difficile			
Replicate 1	210.36	210.15	0.21
Replicate 2	223.47	249.63	0
Replicate 3	196.90	173.27	23.63
Replicate 4	181.72	182.44	0
C. scindens			
Replicate 1	121.67	44.48	77.19
Replicate 2	142.03	22.35	119.68
Replicate 3	93.38	16.17	77.20
Replicate 4	78.54	10.79	67.75
C. difficile and C. scindens			
Replicate 1	153.77	54.25	99.51
Replicate 2	169.30	42.09	127.20
Replicate 3	126.16	17.97	108.19
Replicate 4	136.51	42.29	94.22

Supplementary References

1. Heap JT, Pennington OJ, Cartman ST, Carter GP, Minton NP. 2007. The ClosTron: a universal gene knock-out system for the genus *Clostridium*. J Microbiol Methods. 70: 452-464
2. Morris GN, Winter J, Cato, EP, Ritchie, AE, Bokkenheuser, VD. 1985. *Clostridium scindens* sp. nov., a human intestinal bacterium with desmolytic activity on corticoids. Int. J. Syst. Bacteriol. 35: 478-481.
3. Hussain HA, Roberts AP, Mullany P. 2005. Generation of an erythromycin-sensitive derivative of *Clostridium difficile* strain 630 (630Deltaerm) and demonstration that the conjugative transposon Tn916DeltaE enters the genome of this strain at multiple sites. J Med Microbiol. 54:137-141.
4. O'Connor JR, Lyras D, Farrow KA, Adams V, Powell DR, Hinds J, Cheung JK, Rood JI. 2006. Construction and analysis of chromosomal *Clostridium difficile* mutants. Mol Microbiol 61:1335–1351.
5. Stabler RA, He M, Dawson L, Martin M, Valiente E, Corton C, Lawley TD, Sebaihia M, Quail MA, Rose G, Gerding DN, Gibert M, Popoff MR, Parkhill J, DouganG, Wren BW. 2009. Comparative genome and phenotypic analysis of *Clostridium difficile* 027 strains provides insight into the evolution of a hypervirulent bacterium. Genome Biol. 10(9):R102.
6. Kurka H, Ehrenreich A, Ludwig W, Monot M, Rupnik M, Barbut F, Indra A, Dupuy B, Liebl W. 2014. Sequence similarity of *Clostridium difficile* strains by analysis of conserved genes and genome content is reflected by their ribotype affiliation. PLoS One. 9:e86535.

7. Kint N, Janoir C, Monot M, Hoys S, Soutourina O, Dupuy B, Martin-Verstraete I. 2017. The alternative sigma factor σ(B) plays a crucial role in adaptive strategies of *Clostridium difficile* during gut infection. Environ Microbiol. 19:1933-1958.
8. Antunes A, Martin-Verstraete I, Dupuy B. 2011. CcpA-mediated repression of *Clostridium difficile* toxin gene expression. Mol Microbiol. 79:882-899.
9. Pereira FC, Saujet L, Tomé AR, Serrano M, Monot M, Couture-Tosi E, Martin-Verstraete I, Dupuy B, Henriques AO. 2013. The spore differentiation pathway in the enteric pathogen *Clostridium difficile*. PLoS Genet. 9:e1003782.
10. Saujet L, Monot M, Dupuy B, Soutourina O, Martin-Verstraete I. 2011. The key sigma factor of transition phase, SigH, controls sporulation, metabolism, and virulence factor expression in Clostridium difficile. J Bacteriol. 193:3186-3196.
11. Wydau-Dematteis S, El Meouche I, Courtin P, Hamiot A, Lai-Kuen R, Saubaméa B, Fenaille F, Butel MJ, Pons JL, Dupuy B, Chapot-Chartier MP, Peltier J. 2018. Cwp19 Is a Novel Lytic Transglycosylase Involved in Stationary-Phase Autolysis Resulting in Toxin Release in *Clostridium difficile*. MBio. 9. pii: e00648-18.
